# Excitonic energy transfer in red algal Photosystem I reveals an evolutionary bridge between cyanobacteria and plants

**DOI:** 10.1073/pnas.2530661123

**Published:** 2026-07-21

**Authors:** Mengyuan Cui, Zihui Liu, Miriam Izzo, Junhua Zhou, Enhu He, Vandana Tiwari, Petar H. Lambrev, R. J. Dwayne Miller, Joanna Kargul, Fulu Zheng, Ajay Jha, Hong-Guang Duan

**Affiliations:** ^a^ Department of Physics, School of Physical Science and Technology, Ningbo University, Ningbo 315211, People’s Republic of China; ^b^ Solar Fuels Laboratory, Center of New Technologies, University of Warsaw, Warsaw 02-097, Poland; ^c^ Stanford PULSE Institute, SLAC National Accelerator Laboratory, Menlo Park, CA 94025; ^d^ Department of Chemical Science, Linac Coherent Light Source, SLAC National Accelerator Laboratory, Menlo Park, CA 94025; ^e^ Biological Research Centre, Szeged 6726, Hungary; ^f^ Departments of Chemistry and Physics, University of Toronto, Toronto, ON M5S 3H6, Canada; ^g^ Zhejiang Key Laboratory of Advanced Optical Functional Materials and Devices, Ningbo University, Ningbo 315211, China; ^h^ Rosalind Franklin Institute, Harwell, Oxfordshire OX11 0QX, United Kingdom; ^i^ Department of Pharmacology, University of Oxford, Oxford OX1 3QT, United Kingdom

**Keywords:** excitonic energy transfer, ultrafast nonlinear spectroscopy, two-dimensional electronic spectroscopy, quantum dissipative system, photosynthetic energy transport

## Abstract

Photosystem I (PSI) converts light into chemical energy with near-unity quantum efficiency, yet its energy-transfer and charge-separation mechanisms remain debated. Evolution has diversified PSI architectures: Cyanobacterial PSI trimers confine red-shifted pigments to the core, whereas plant PSI-Light Harvesting Complex Isupercomplexes incorporate extensive peripheral red and charge-transfer states that reshape trapping. The unicellular red alga *Cyanidioschyzon merolae* exemplifies functional diversification across distinct evolutionary branches, combining a photosystem II and plant-like monomeric PSI core associated with a varying number of light harvesting antenna subunits, Light Harvesting Complexes from Red Lineage (LHCR). This hybrid organization functionally bridges mechanistic models across different lineages. We applied two-dimensional electronic spectroscopy at ultralow temperatures (8 and 80 K) to disentangle overlapping excitation pathways in *C. merolae* PSI. Cryogenic measurements suppressed thermal broadening, resolving five dynamical components: subpicosecond equilibration (0.3 to 0.8 ps) across the core–LHCR interface, subsequent population transfer (2.6 to 4 ps) into progressively lower-energy manifolds, and slower feeding (18 to 53 ps) into red pools distributed across both core and antenna. On the longest timescales (hundreds of ps), a persistent ground-state bleach signifies excitons stabilized in terminal sinks. Notably, comparison of 8 K and 80 K spectra reveals that excitations are heterogeneously partitioned among multiple sinks at low disorder, whereas modest thermal activation (kT ∼ 55 cm^−1^) promotes selective convergence into core-associated red chlorophylls. Atomistic excitonic modeling with time-nonlocal master equations supports these observations, revealing temperature-dependent energy redistribution. Overall, *C. merolae* PSI expands the kinetic funnel by distributing trapping sites, enhancing spectral coverage while maintaining high efficiency, which is an important functional diversification during evolution.

## Significance

Photosystem I (PSI) converts sunlight into chemical energy with remarkable efficiency, yet how its energy-transfer pathways evolved across cyanobacteria, alga, and plants remain unclear. Using ultralow-temperature two-dimensional electronic spectroscopy combined with quantum-dynamical modeling, we show that PSI from the red alga *Cyanidioschyzon merolae* contains two spatially distinct low-energy “sinks” located in both the core and antenna. Unlike cyanobacteria (core-dominated) or land plants (antenna-dominated), this intermediate architecture redistributes excitation energy in a temperature-dependent manner while maintaining efficient trapping for optimal energy conversion. Our results reveal how antenna expansion reshaped energy flow during evolution without compromising performance. These insights establish general design principles for balancing efficiency and resilience in natural and artificial light-harvesting systems.

**Fig. 1.**
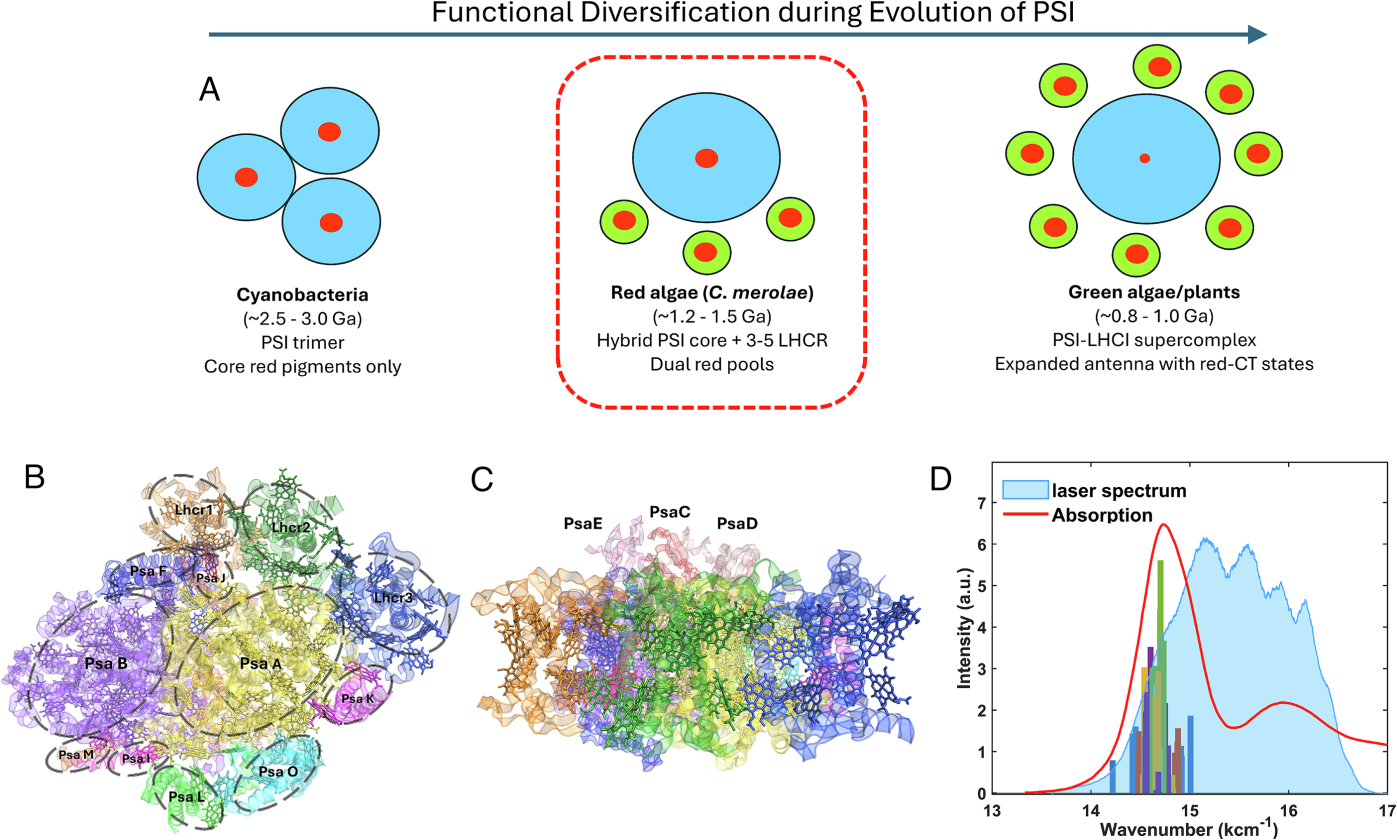
Evolutionary context and spectral characterization of PSI. (*A*) Cartoon schematic illustrating the structural evolution of Photosystem I (PSI) from cyanobacterial trimers with core-localized red chlorophylls, through the red alga *C. merolae* intermediate featuring a monomeric core plus a small number of LHCR subunits representing dual red pools, to plant PSI-LHCI supercomplexes. In the plant system, while a small number of red chlorophylls remain in the core (indicated by smaller red markers), their contribution to excitation trapping is relatively weak, with the dominant red states arising from the antenna. (*B* and *C*) Top and side views of *C. merolae* PSI, respectively, highlighting core subunits (PsaA/B) and peripheral Lhcr1-3, as well as bridging subunit PsaO. (*D*) Steady-state absorption spectrum (red line) of PSI compared with the excitation laser spectrum (blue area), showing broad pigment absorption with overlap to the experimental excitation bandwidth.

Photosystem I (PSI) is a fundamental component of the light-dependent reactions of oxygenic photosynthesis (1, 2). Present in cyanobacteria, alga, and plants, this multisubunit pigment-protein supercomplex exhibits near-unity quantum efficiency in charge separation (CS) process after solar energy absorption (3–6). The extraordinary performance of PSI arises from its intricate network of pigment molecules, primarily chlorophyll (Chl) a, embedded within a protein scaffold that orchestrates the absorption, migration, and trapping of photoexcitation energy. These pigment arrays act cooperatively, mediating excitation energy transfer (EET) across distances of several nanometers with subpicosecond to picosecond timescales, ultimately leading to primary CS in the reaction center (RC) and electron transfer to downstream cofactors (1, 7–9). Despite this exceptional performance, the precise sequence of excitonic energy transfer, trapping, and charge separation remains a subject of debate. A unified picture has been elusive in part because PSI has diversified considerably during evolution, acquiring distinct antenna systems in cyanobacteria, red alga, and green alga/plants, as depicted in Fig. 1*A* (10–12). Resolving how these evolutionary changes remodel the excitation landscape requires integrating structural data with ultrafast spectroscopic measurements.

In cyanobacteria such as *Synechocystis sp. PCC 6803* and *Synechococcus sp. PCC 7002*, PSI typically assembles as a trimer, each monomer containing ∼95 chlorophyll a molecules and ∼20 carotenoids arranged within a conserved core (3, 13). A subset of low-energy “red” chlorophylls absorb beyond 700 nm, forming energetic sinks that bottleneck transfer into the RC (14–18). Two-dimensional electronic spectroscopy (2DES) studies of cyanobacterial PSI trimers revealed sub-100 fs equilibration among bulk core pigments, followed by ps-scale population of the red pools, which govern the overall trapping timescale (19). Although the number and spectral position of these red pigments vary across species, their role as the dominant kinetic choke point is conserved.

In land plants, PSI exists as a monomeric core surrounded by a belt of 8-10 Light Harvesting Complex I (LHCI) (Lhca) heterodimers, roughly doubling the pigment count to ∼200 Chls per complex (5). While the PSI core still contains a small number of red chlorophylls, their spectral contribution and trapping strength are relatively modest compared to antenna-associated red and charge-transfer states. The dominant low-energy states arise from LHCI subunits (particularly Lhca1/4), which absorb at longer wavelengths (∼730 to 740 nm) and act as the primary kinetic traps (1, 20, 21). Thus, although core red chlorophylls are present, they play a secondary role in excitation trapping relative to antenna-based states. 2DES studies of intact PSI-Light Harvesting Complex I (PSI-LHCI) supercomplexes showed ultrafast (<0.5 ps) equilibration in the core, ∼3 to 4 ps transfer into LHCI red/CT states, and slower tens-of-ps components reflecting delayed release from these states (22–27). Thus, while the plant PSI expands spectral coverage, it also introduces more heterogeneous relaxation pathways, illustrating how antenna enlargement alters dynamics without compromising overall efficiency.

The unicellular red alga *Cyanidioschyzon merolae* retains a combination of cyanobacterial-like core organization and eukaryotic antenna features, making it a valuable system for understanding how PSI architecture evolved and diversified. Structural studies show that its PSI is monomeric, like in plants, but binds only 3-5 LHCR subunits (each with ∼11 to 13 Chls and multiple zeaxanthins), yielding an intermediate pigment count of ∼130 to 160 Chls (28). A distinctive feature is the incorporation of the eukaryotic subunit PsaO, which coordinates three Chls at the antenna–core interface. The core also retains cyanobacterial-like subunits such as PsaM, producing a hybrid organization. This architecture differs from both cyanobacteria and plants in two key respects. First, low-energy pigments are distributed across both the core and LHCR, creating dual sinks. Second, pigment bridges formed by PsaO and interfacial LHCR Chls enable equilibration across the core–antenna boundary, in contrast to the slower transfer observed in plants. Functionally, *C. merolae* PSI thus represents an evolutionary intermediate, extending absorption through LHCR while maintaining a cyanobacterial-like trapping arrangement. Despite its significance, *C. merolae* PSI has not been systematically explored using multidimensional spectroscopy. Most prior ultrafast work has focused on cyanobacterial cores or plant PSI-LHCI supercomplexes, with isolated Lhca subunits also studied at cryogenic temperatures (19, 29, 30). At low temperatures, excited state red Chl population occurs in 4 to 6 ps, slower than at room temperature, reflecting a general deceleration of EET pathways in photosynthetic systems (31–35). These studies highlighted the central role of low-energy pigments, whether core-localized red Chls, LHCI CT states, or both in shaping PSI kinetics. Yet the absence of a red algal 2DES study has left a critical gap between prokaryotic and eukaryotic paradigms.

Here, we address this gap by applying 2DES to PSI from *C. merolae*. By combining ultralow-temperature 2DES measurements (8 and 80 K) complemented with dynamics calculations using time-nonlocal master equations and atomistic excitonic Hamiltonians, we resolve overlapping excitation pathways in PSI. This strategy reveals ultrafast equilibration across the core–antenna interface, picosecond population of low-energy states, and slower transfer into the RC. If low-energy traps were not redistributed during antenna expansion, excitations would accumulate in a limited number of sites, increasing the likelihood of kinetic bottlenecks, recombination losses, or delayed delivery to the reaction center. By contrast, in *C. merolae*, the low-energy manifold is distributed across both antenna and core, generating multiple accessible equilibration pathways. This organization broadens the low-energy landscape while remaining compatible with the high overall trapping efficiency characteristic of PSI, but it also introduces kinetic heterogeneity because escape from red states is partly thermally activated. By directly comparing these dynamics with cyanobacterial and plant PSI, we demonstrate that *C. merolae* PSI serves as a mechanistic and functional evolutionary bridge, revealing how incremental antenna expansion and redistribution of low-energy pigments reshaped energy transfer and trapping during the functional diversification of oxygenic photosynthesis.

## Results and Discussion

The PSI–LHCR complexes were purified from genetically engineered *C. merolae* strains expressing a His_6_-tagged PsaD subunit, and their structural integrity, pigment composition, and photochemical activity were confirmed through biochemical characterization. Detailed methods and validation data, including absorption spectroscopy, SDS-PAGE analysis, and oxygen consumption assays, are presented in *SI Appendix*, Figs. S1 and S2. A structural overview of the PSI–LHCR complex is provided in Fig. 1 *B* and *C*, shown from the top and side views. The core RC subunits, PsaA and PsaB, which host the special pair of Chls responsible for primary charge separation, are highlighted at the center of the complex. Surrounding the RC are a series of small core subunits, including PsaF, PsaJ, and PsaK, which play critical roles in mediating energy transfer between the RC and the peripheral antenna. The LHCR antenna complexes: Lhcr1, Lhcr2, and Lhcr3 are positioned asymmetrically around the core, partially surrounding it and forming a modular light-harvesting belt. These subunits contribute additional pigments that extend the absorption cross-section of PSI and provide low-energy exciton states that are funneled toward the RC.

The steady-state absorption spectrum of the *C. merolae* PSI–LHCR complex is shown in Fig. 1*D* (red solid line). As expected for red algal PSI, the spectrum exhibits distinct features characteristic of chlorophyll a, with prominent peaks centered near 630 nm and 680 nm. For comparison, the spectral profile of the excitation laser pulses employed in our 2DES measurements is overlaid as a blue shadow. The laser spectrum covers both chlorophyll absorption maxima, demonstrating that our experimental configuration allows us to simultaneously excite higher-energy pigments absorbing near 630 nm together with the bulk antenna states absorbing around 680 nm. This overlap ensures that the full excitonic manifold contributing to energy transfer in PSI is effectively accessed in our ultrafast measurements.

### Two-Dimensional Electronic Spectroscopy of PSI Complex.

**Fig. 2.**
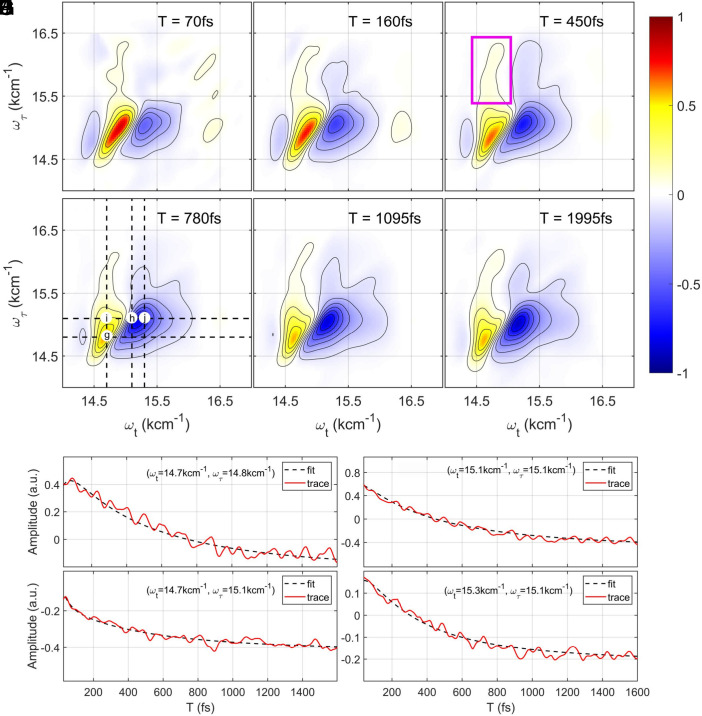
Two-dimensional electronic spectra of PSI at 8 K. Panels (*A*–*F*) show total real spectra at selected waiting times (70 to 1,995 fs). Red (positive) features arise from ground-state bleach and stimulated emission, while blue (negative) features correspond to excited-state absorption. The persistence of overlapping red/blue peaks reflects strong excited-state absorption between single and double excited states, while the emergence of cross peaks (magenta box) illustrate downhill transfer pathways. (*G*–*J*) Time-dependent traces of selected diagonal and cross peaks (red) compared with global fits (black dashed), validating the kinetic model used to extract transfer timescales.

The 2DES has emerged as a unique probe to disentangle different excited state relaxation pathways, offering simultaneous femtosecond temporal resolution and high spectral selectivity (36–44). Unlike linear absorption or pump–probe spectroscopy, 2DES separates excitation and detection frequencies, enabling the direct observation of energy-transfer events via off-diagonal cross-peaks and allowing the tracking of coherent phenomena through oscillatory modulations in signal amplitudes (44–46). Following steady-state characterization, we performed 2DES on the *C. merolae* PSI–LHCR complex at 8 K, where suppressed thermal motion sharpens transitions and enhances the visibility of cross-peaks. Representative real-part 2D spectra at waiting times (T) of 70, 160, 450, 780, 1095, and 1995 fs are shown in Fig. 2*A*–*F*. In these spectra, positive signals (red) correspond to ground-state bleaching (GSB) and stimulated emission (SE), whereas negative signals (blue) reflect excited-state absorption (ESA). Measurements at T = 0 fs were omitted because of pulse overlap artifacts that prevented reliable separation of absorptive and dispersive components. At T = 70 fs (Fig. 2*A*), a strong composite feature is observed near excitation ∼14,500 cm^−1^ and detection ∼16,500 cm^−1^. Here, positive GSB/SE overlaps with negative ESA, reducing the apparent bleach amplitude. This overlap reflects strong coupling between pigments, where excitation of bulk chlorophylls feeding into higher-energy states simultaneously gives rise to induced absorption. Above ∼15,500 cm^−1^ on the detection axis, the spectra display pre-dominantly ESA, consistent with access to higher-lying excitonic manifolds. Notably, at this low temperature, several off-diagonal cross-peaks are clearly resolved (highlighted in Fig. 2*C*), indicating direct downhill transfer channels from bulk antenna pigments into lower-energy states, including the red pools associated with both LHCR and core pigments. By 160 fs (Fig. 2*B*), the overall line shape remains dominated by overlapped red-blue features, though the ESA contribution increases in relative strength. This suggests progressive population of excited states that participate in absorption from already occupied levels. The coexistence of GSB and ESA persists through 2 ps (Fig. 2*A*–*F*), indicating that low-energy excitations are populated early but remain dynamically coupled to higher-energy states, a hallmark of strongly delocalized excitonic networks.

To probe these dynamics, we extracted time-domain traces from two diagonal and two cross-peaks marked in Fig. 2*D*. Diagonal peaks monitor excitations that remain on initially excited bulk states, whereas below-diagonal cross-peaks report population transfer to lower-energy acceptors. For example, the feature linking excitation near 15,000 cm^−1^ with detection at ∼14,700 cm^−1^ (Fig. 2*G*) grows within the first few hundred femtoseconds, consistent with rapid downhill transfer from bulk antenna pigments to red-shifted sinks. The diagonal trace at ∼15,100 cm^−1^ (Fig. 2*H*) shows corresponding decay, reflecting depletion of high-energy populations. Together, these pairs of diagonal and cross-peak dynamics provide direct spectroscopic evidence for population flow from bulk chlorophylls to progressively redder states. To quantify these behaviors, we employed a global fitting approach (47) (*SI Appendix*). This analysis applies a common set of exponential components across all traces, yielding fits (black dashed lines in Fig. 2*G*–*J*) that closely reproduce the experimental kinetics. The excellent agreement confirms that the observed decays and rises can be captured by a shared set of dynamical processes, spanning sub-100 fs equilibration through multipicosecond population of red pigments. While specific time constants are discussed later, at this stage the analysis demonstrates that the spectral signatures of bulk-to-red transfer are robust and reproducible across multiple probe regions. Extending the waiting time to hundreds of picoseconds (*SI Appendix*, Fig. S11) reveals a marked simplification of the spectra. By ∼100 ps, the ESA contributions vanish entirely, leaving only positive bleach features corresponding to the lowest exciton states. This transition indicates that excitations have fully relaxed into long-lived red sinks, which persist as ground-state bleach signals after excited-state absorption pathways have decayed. The persistence of these GSB signals highlights the stability of the lowest-energy pigments, which act as terminal sites for energy funneling prior to charge separation.

**Fig. 3.**
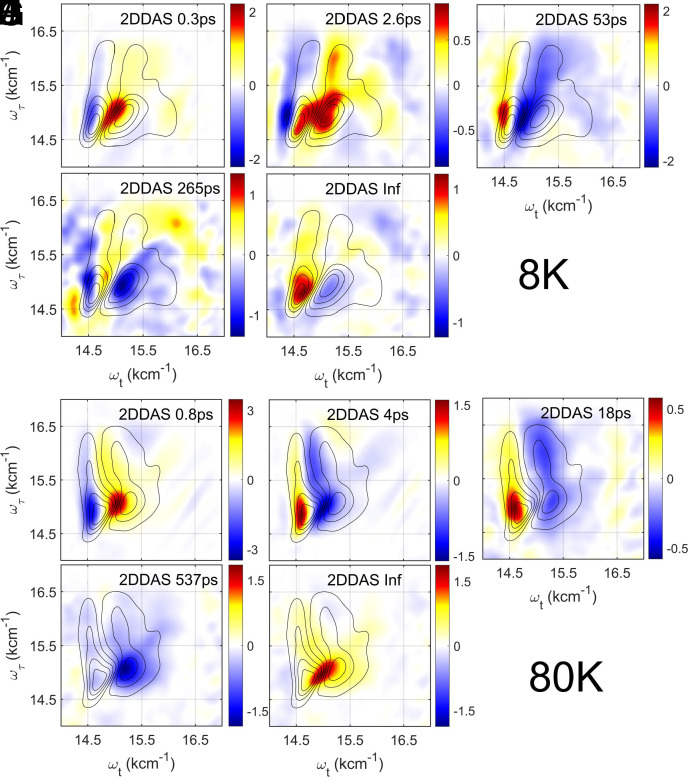
Decay-associated spectral (DAS) components from global analysis of PSI at 8 K (*A*–*E*) and 80 K (*F*–*J*). Heat maps show the amplitudes of the 2D-DAS, where positive features (red/yellow) correspond to decaying components and negative features (blue) correspond to rising components with the indicated lifetimes. Overlaid contour lines denote the corresponding experimental real 2DES signal to guide comparison with the underlying spectral regions. From (*A*–*E*) and from (*F*–*J*), we selected the contour lines near T = 10 ps at temperatures of 8 K and 80 K, respectively. (*A*) The 0.3 ps component captures ultrafast downhill transfer within the antenna. (*B*) The 2.6 ps component reflects further equilibration and depletion of bulk chlorophyll states along the diagonal. (*C*) The 53 ps contribution highlights population transfer into red pools across both core and LHCR. (*D*) The 265 ps component indicates progressive accumulation of long-lived excitations in these sinks. (*E*) The infinite component displays the excitons stabilized in the lowest-energy states. (*F*–*J*) The 2D-DAS with the corresponding resolved decay time constant at 80 K. The fastest DAS component shows the lifetime of 0.8 ps. The other components belong to the timescale of 4 ps, 18 ps, 537 ps, and infinity, respectively.

Application of the global fitting procedure resolved five decay-associated spectral (2DDAS) components at 8 K and 80 K, with timescales at 8 K spanning from 0.3 ps to an effectively nondecaying contribution. These maps are shown in Fig. 3*A*–*E*, with contours of the original 2DES spectra overlaid to highlight the involved regions. The fastest component (0.3 ps; Fig. 3*A*) is characterized by a strong positive-negative pair centered around ($\omega_{\tau }$, $\omega_{t}$) = (15,500, 14,500) cm^−1^, which we attribute to ultra- fast downhill energy transfer from higher-lying antenna pigments into lower-energy states, thereby minimizing losses while maintaining directional energy flow. This subpicosecond equilibration reflects the rapid redistribution within strongly coupled chlorophyll domains. The following 2.6 ps component (Fig. 3*B*) displays a diagonal bleach feature centered near 15,000 cm^−1^, signifying depletion of bulk antenna states. On longer timescales, the 53 ps component (Fig. 3*C*) exhibits complementary blue signatures at the low-energy side, consistent with population arrival into red pools of both the LHCR and core. The 265 ps contribution (Fig. 3*D*) reveals more pronounced accumulation in these sinks, highlighting their role as long-lived reservoirs. Finally, the effectively infinite component (Fig. 3*E*) retains a persistent red bleach in the ($\omega_{\tau }$, $\omega_{t}$) ≈ (14,500 to 15,000) cm^−1^ region, corresponding to excitations localized in the terminal red chlorophyll states. This enduring signal represents the stabilized precursors to charge separation.

Beyond population dynamics, the 2DES data reveal oscillatory features indicative of vibrational coherence. Such oscillations observed in 2DES studies of different photosynthetic complexes were initially proposed as signatures of coherent excitonic superpositions, implying that wave-like transport might augment classical hopping (48). Subsequent experimental and theoretical studies, however, demonstrated that these signals predominantly arise from vibrational coherences (46, 49–51). To probe these contributions in our system, we analyzed residuals from global fitting and constructed frequency-resolved vibrational maps. The resulting maps (*SI Appendix*, Fig. S6 *A*–*C*) exhibit oscillations at 325, 496, and 753 cm^−1^, aligned along the antidiagonal, consistent with vibrational progressions coupled to the excitonic manifold. Additional maps in *SI Appendix* confirm that multiple coherent modes persist across PSI antenna and reaction center regions. Based on prior spectroscopic assignments, the 325 cm^−1^ band reflects porphyrin-ring deformations and Mg-N bending in Chla (52), the 496 cm^−1^ mode corresponds to skeletal deformations and vinyl-macrocycle bending (53), and the 753 cm^−1^ band to C-C/C-N macrocycle stretching, long recognized as coherence carriers in PSI and LHC proteins (54). Their presence across diagonal and cross-peak regions suggests possible roles in both bulk antenna relaxation and feeding of red pools. While these observations indicate potential vibronic contributions to exciton dynamics, a quantitative description would require explicit treatment of vibrational modes within a non-Markovian framework. Given the system size and computational complexity, such analysis is beyond the present scope, but represents an important direction for future investigation.

### Temperature Dependence of Excitonic Relaxation.

**Fig. 4.**
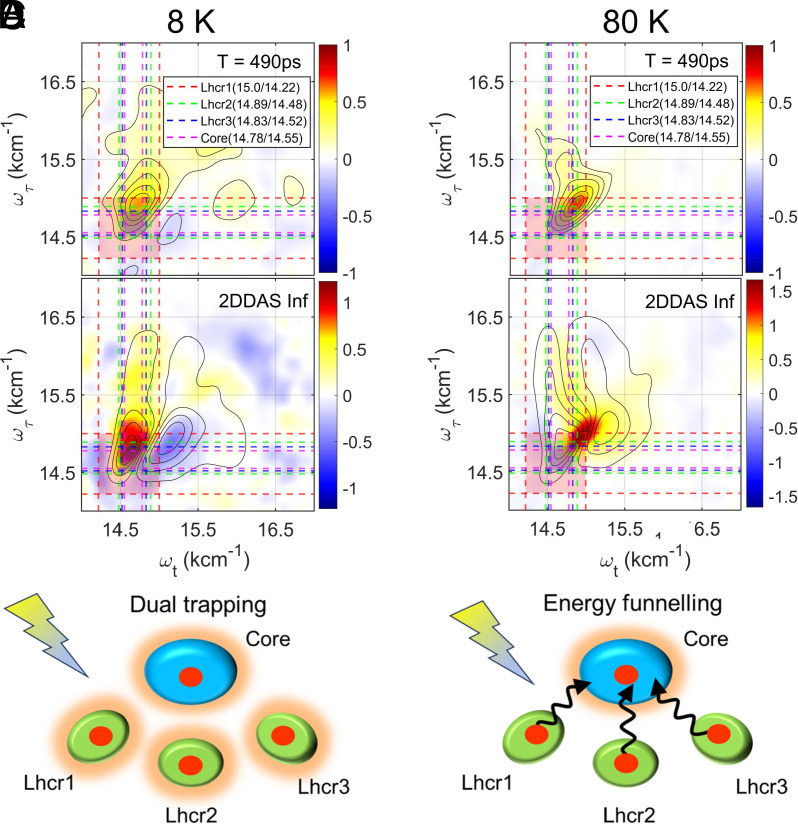
Temperature-dependent dynamics of PSI red pools. Two-dimensional spectra at 8 K (*A*) show broad ground-state bleach features reflecting heterogeneous population of multiple red pools in LHCR and the core. At 80 K (*B*), features sharpen and converge into the core region, consistent with thermally assisted funneling into dominant sinks. Dashed lines mark the site energies of Lhcr1-3 and the core red pigments. Schematics below, (*C* and *D*), illustrate this transition from distributed to thermally guided trapping pathways.

To investigate how temperature modulates excitonic relaxation dynamics, we directly compared 2DES measurements of PSI at 8 K and 80 K (Fig. 4). Full 80 K datasets are available in *SI Appendix*, Figs. S7–S10. At early waiting times (T ∼ 15 ps), both temperatures show characteristic GSB and SE features near the diagonal, accompanied by negative ESA below. Notably, the 2DES spectra reveal robust downhill energy transfer from high-energy Chls into red-shifted acceptors. However, striking differences emerge at later time delays (T ∼ 500 ps). At 8 K, GSB features below 14,600 cm^−1^ persist but are spectrally broad and spatially dispersed across Lhcr1 ∼(15,000/14,220 cm^−1^), Lhcr2 ∼(14,890/14,480 cm^−1^), Lhcr3 ∼(14,830/14,520 cm^−1^), and the core red pool ∼(14,780/14,550 cm^−1^), indicating heterogeneous exciton trapping across multiple sites. In contrast, at 80 K, GSB features sharpen markedly and converge more strongly within the core red region, suggesting selective thermal funneling. The resulted data of excited states of core and antennas have been listed in the supporting Excel file. This interpretation is supported by 2DDAS analysis (Fig. 3*A*–*J*), which resolves comparable kinetic components at both temperatures: sub-ps equilibration (0.3 to 0.8 ps), fast bulk-state depletion (2.6 to 4 ps), red-pool feeding (18 to 53 ps), and long-lived terminal population (≥265 to 537 ps). Yet, the spectral amplitudes of these components differ: 8 K traces show broader decay features and greater spectral congestion, while 80 K signals display narrower, better-resolved transitions into the core sink. These findings indicate that the temperature does not introduce new kinetic phases but redistributes exciton populations: The slower red-pool signals become spectrally sharper at 80 K, reflecting more convergent trapping compared to the heterogeneous sink occupation seen at 8 K. Thus, while the overall energy-transfer pathways are conserved, thermal energy reshapes the exciton trapping landscape by biasing population flow toward specific red-chlorophyll sites.

### Theoretical Modeling of Excitonic Population Dynamics.

To complement the experimental 2DES results and provide a microscopic picture of the exciton relaxation in PSI, we performed quantum-dynamical simulations based on an atomistic excitonic Hamiltonian. The site energies of all Chl-pigments were obtained from ab-initio electronic structure calculations, while excitonic couplings were evaluated within the dipole–dipole approximation. The full details of these calculations and the resulting Hamiltonian are provided in Dataset S1. Together, these parameters define the electronic landscape across the PSI core and LHCR antenna complexes, enabling us to probe how strongly coupled pigments form delocalized excitonic states. Based on this framework, the low-energy (red) excitonic states were identified from the calculated site energies and excitonic eigenstates. These states arise from pigments experiencing pronounced red-shifted site energies due to their local protein environments and interpigment interactions, and are distributed across both the PSI core and LHCR antenna complexes. Red states were defined as chlorophylls exhibiting site energies corresponding to wavelengths longer than 685 nm, consistent with their role as low-energy exciton sinks. Based on this criterion, a total of 17 red chlorophylls were identified. The spectral positions of these red states were further validated by comparison between the calculated absorption spectrum, obtained using the time-nonlocal response formalism, and the experimental steady-state absorption. In addition, the identified energy ranges are consistent with previously reported long-wavelength chlorophyll forms in PSI systems (55–57). In the PSI–LHCR complex of *C. merolae*, the red excitonic states are found to be located around ∼703 nm and within the 685 to 688 nm range. The spatial distribution and corresponding spectral positions of these states are illustrated in *SI Appendix*, Fig. S5 and further described in *SI Appendix*, section III, providing direct support for their assignment to both core and antenna subunits.

**Fig. 5.**
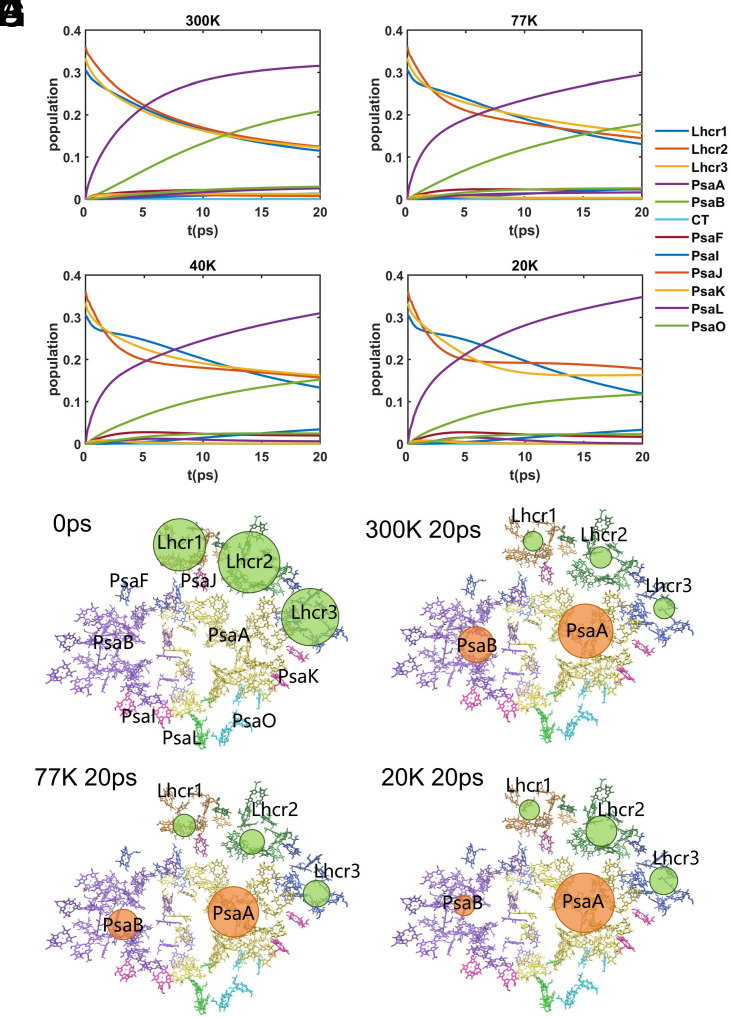
Theoretical modeling of exciton dynamics in PSI subunits. (*A*–*D*) Population dynamics simulated with a time-nonlocal quantum master equation using Hamiltonians derived from ab-initio site energies and dipole–dipole couplings at 300 K, 77 K, 40 K, and 20 K. Calculations reveal rapid equilibration within LHCRs and the PSI core (sub-ps to few ps), followed by slower population of low-energy red states. (*E*–*H*) Structural maps, with the size of coloured circles to represent relative population, illustrate population flow from antenna (Lhcr1–3) into distributed sinks within the core (PsaA/B) over 20 ps at different temperatures.

The subsequent energy-transfer dynamics were computed within the framework of a time-nonlocal (TNL) quantum master equation. In this approach, each pigment is modeled as a two-level system coupled to a structured phonon bath that mimics protein-induced fluctuations. The bath was described as a set of harmonic oscillators with spectral densities chosen to reproduce both fast intramolecular vibrations and slower protein motions, as established in prior work on PSI and related complexes. This system-bath formalism captures both dissipative relaxation and coherent oscillatory contributions, making it particularly well-suited to interpret the experimentally observed vibrational coherences. Compared to simpler rate-equation models, the TNL method retains non-Markovian memory effects, allowing us to account for transient coherence and the role of vibronic mixing. (Fig. 5*A*–*D*) presents the simulated population dynamics across PSI subunits at different temperatures (300 K, 77 K, 40 K, and 20 K), obtained using the TNL master equation and assuming uniform initial excitation of all three LHCR complexes. The calculations reveal rapid equilibration within LHCR units, occurring within the first picosecond, in close agreement with the experimentally resolved ∼100 fs to 1 ps components. On longer timescales, excitations redistribute toward both the core (PsaA/B) and low-energy red pools located in LHCR and the core, consistent with the dual-sink architecture of *C. merolae* PSI. Importantly, the calculations capture a clear temperature dependence, with slower population transfer and enhanced accumulation in low-energy states at reduced temperatures, in qualitative agreement with experimental observations. Multiexponential analysis of the simulated traces yields characteristic timescales ranging from sub-ps equilibration to ∼20 ps red-state feeding, consistent with the experimentally resolved decay-associated spectral components. The extracted timescales and amplitudes are summarized in *SI Appendix*, Table II, along with a direct comparison to experimental values across temperatures.

## Discussion

Our results offer a coherent mechanistic picture of excitation-energy transfer in PSI from *C. merolae*, a species that holds a key evolutionary position between cyanobacteria and the green lineage. By integrating cryogenic 2DES with room-temperature quantum-dynamical simulations, we resolve how energy is redistributed across pigments and clarify the functional role of red chlorophylls. A central observation in this work is the recognition that *C. merolae* PSI employs a dual-sink arrangement, with low-energy pigments located both within the core and the LHCR antennae. Our temperature-dependent 2DES results provide a critical perspective on the functional relevance of dual-sink trapping in *C. merolae* PSI. Although these extremophilic red alga never experience cryogenic conditions, measurements at 8 K reveal how excitations distribute when thermal motion is essentially frozen (kT ∼5.5 cm^−1^). Under these conditions, excitations remain in local minima, yielding broad ground-state bleach signals that directly expose the heterogeneity of red chlorophyll traps across both LHCR and the core. At elevated cryogenic and physiological temperatures, however, thermal activation becomes significant: kT ∼55 cm^−1^ at 80 K and ∼208 cm^−1^ at 300 K are comparable to the energy gaps between antenna reds (e.g., Lhcr1 at ∼14,220 cm^−1^) and core red chlorophylls (∼14,550 cm^−1^). Thus, even a modest increase in temperature enables excitations to overcome uphill gaps of 100 to 300 cm^−1^ and converge into the core-associated sinks. Importantly, this does not require each exciton to carry the full energy difference; rather, thermal disorder in the protein matrix provides a distribution of phonon modes and entropic fluctuations that occasionally supply sufficient quanta to bridge the gap. In this way, dynamic sampling of vibrational environments ensures that even energetically “trapped” excitations can be liberated and redirected toward the reaction center at physiologically relevant temperatures. Functionally, this means that under physiological conditions PSI behaves as a single effective funnel to P700, while the apparent “dual-sink” behavior is suppressed. Nonetheless, the existence of multiple red traps is not merely vestigial. They expand the spectral range of light harvesting and provide backup pathways under conditions where the RC is saturated or transiently closed, ensuring both robustness and efficiency in PSI energy conversion (58).

Under conditions in which the reaction center is less accessible, the distributed red manifold is expected to alter how excitations equilibrate across PSI, but not to store excitation over the timescale of reaction-center reopening. In PSI, population transfer from red states typically occurs on picosecond to few-hundred-picosecond timescales, extending to nanosecond components mainly under cryogenic or closed-RC conditions, whereas reopening of the reaction center and long-lived charge-separated states occurs on much slower microsecond-to-millisecond timescales. We therefore interpret the red states not as long-term storage sites, but as short-lived low-energy waystations that broaden the absorption range and transiently redistribute excitation within the intrinsic trapping window. In this context, direct photoprotection in PSI is more appropriately linked to established mechanisms such as P700 oxidation and control of acceptor-side limitation.

This interpretation is reinforced by our quantum-dynamical simulations at room temperature, which reproduce the experimental timescales and reveal how excitations partition between core and LHCR sinks. The theory indicates that equilibration is accelerated at physiological conditions. Overall, such a mechanism of dual trapping and selective funneling is absent in cyanobacteria, where a single core-localized red pool dictates dynamics, and in plants, where long-lived LHCI charge-transfer states dominate. *C. merolae* thus represents a true functionally evolutionary midpoint: retaining the efficiency of core-based trapping while introducing antenna-associated sinks that expand functional diversity. Altogether, these findings show that redistribution of low-energy states during antenna expansion has conditional rather than universal functional consequences. Under physiological temperatures, thermal activation allows excitations to escape antenna-associated red states and ultimately converge toward the reaction center, preserving an effective directional funnel. Under lower temperatures, the same states become quasi-traps and generate greater kinetic heterogeneity. The role of the distributed red manifold is therefore to broaden spectral coverage and diversify equilibration pathways, instead of providing a dedicated quenching channel. This balance also explains why such an architecture is not universal: Stronger or more numerous red states can improve light capture under far-red or light-limited conditions, but they also impose thermally activated bottlenecks that slow trapping. PSI architectures thus reflect lineage-specific ecological compromises rather than a single globally optimal design principle.

## Conclusion

In this study, we have combined 2DES with theoretical modeling to disentangle the excitonic dynamics of PSI from *C. merolae*, an organism occupying a pivotal evolutionary niche between cyanobacteria and plants. By undertaking measurements under cryogenic conditions, thermal disorder was sufficiently suppressed to resolve otherwise overlapping spectral features, permitting identification of discrete kinetic components and clarification of how excitation energy is channelled through both antenna and core pigments. Global analysis revealed five characteristic timescales, from subpicosecond equilibration within LHCR subunits to tens-of-picoseconds population transfer into low-energy red pools. Crucially, the results demonstrate that *C. merolae* PSI sustains dual excitonic sinks distributed across LHCR and the core, in contrast to the strictly core-localized red pools of cyanobacteria and the LHCI-dominated sinks of higher plants. Temperature-dependent measurements further illuminate the functional implications of this architecture. At 8 K, excitations remain heterogeneously distributed across multiple low-energy sites, directly exposing the inherent static disorder of the red pools. By 80 K, however, modest thermal activation (kT ∼55 cm^−1^) is already sufficient to overcome some of the uphill gaps of 100 to 300 cm^−1^ between antenna and core pigments, thereby biasing trapping into the core-associated sinks. At physiological temperatures, where kT approaches ∼200 cm^−1^, entropic fluctuations of the protein–phonon environment ensure that such barriers are readily surmounted, producing a single effective funnel into the reaction center. Thus, the dual-sink architecture is best viewed as a lineage-specific compromise rather than a universally optimal solution. Red chlorophylls broaden the usable absorption range and create a distributed low-energy manifold that transiently partitions excitations on the intrinsic picosecond trapping timescale. At physiological temperatures, thermal activation largely restores an effective funnel toward the reaction center, whereas at lower temperatures the same manifold produces more heterogeneous equilibration and slower release from red states. Our results therefore support spectral expansion and conditional kinetic flexibility, but not long-term exciton storage during reaction-center closure. In this sense, *C. merolae* provides insight into a mechanistically intermediate PSI architecture linking core-dominated cyanobacterial systems with the more antenna-dominated low-energy landscapes of plant PSI.

## Materials and Methods

### Sample Preparation.

The genetic strategy to introduce the modified His_6_-tagged psaD construct (CMV144CT, 417 bp) into the URA locus of the *C. merolae* M4 mutant was adapted from the protocol of Fujiwara et al. (59). The biochemical characterization of the recombinant PSI complex for assessment of purity, structural integrity, and photochemical activity of the biophotocatalyst, in comparison with the native PSI complex, are reported in Izzo et al. (60). The engineered *C. merolae* strain was cultivated in Allen 2 medium (pH 2.5) at 42 ^°^C under continuous white light illumination (90 μmol photons·m^−2^·s^−1^; Panasonic FL40SS-ENW/37) with gentle bubbling with 3 to 5% CO_2_ in air, as previously described in refs. 61 and 62. Cultures (9 L) were grown to an OD680 of 0.9 to 1.0 for isolation of thylakoids and PSI purification, as described in Haniewicz et al. (62). For thylakoids isolation, cells were disrupted on ice with 0.1 mm glass beads for 13 cycles (10 s “on,” 4 min “off”) in buffer A (10 mM CaCl_2_, 5 mM MgCl_2_, 25% (w/v) glycerol, 40 mM MES-NaOH, pH 6.1) supplemented with DNase I (5 mg), RNase (10 μL), and a protease inhibitor cocktail (Thermo Fisher) (1 tablet per 50 mL). The homogenate was filtered (Whatman paper, a premium, highly standardized brand of cellulose microfiber filter paper and the global benchmark in scientific laboratories for accurate filtration, separation, and sample preparation), then centrifuged at 180,000×g for 25 min at 4 ^°^C to pellet thylakoids. Thylakoid membranes were washed three times with buffer A, resuspended to final Chl a concentration of 2 to 5 mg·mL^−1^, then snap-frozen in liquid nitrogen for further use.

The His_6_-PsaD-PSI complex was purified, as reported in Izzo et al. (60). Briefly, the His-tagged complex was purified using immobilized metal affinity chromatography (IMAC). Thylakoids (1 mg Chl a·mL^−1^ were solubilized with 1% (w/v) n-dodecyl-β-D-maltoside (DDM) in the dark on ice for 40 min. All the subsequent procedures were performed at 4 ^°^C under dim green light. The solubilized fraction was filtered and loaded onto a 1 mL HisTrap HP column (Cytiva), pre-equilibrated with 5 column volumes (CV) of the wash buffer (3 mM CaCl2, 0.03% DDM, 25% glycerol, 20 mM imidazole, 40 mM HEPES-NaOH, pH 8.0). After washing with 10 CV of the wash buffer, bound PSI complex was eluted using a linear imidazole gradient (0 to 1 M) in the same buffer. Eluted fractions were analyzed via UV-VIS absorbance spectroscopy (Shimadzu UV-VIS 1800) (*SI Appendix*, Fig. S1). Imidazole was removed by buffer exchange using Vivaspin-20 concentrators (100 kDa MWCO, Sartorius) at 4,000×g, 4 for 15 min. Elution buffer was diluted 1:1 with wash buffer without imidazole and the exchange repeated two more times.

### 2D Electronic Measurements with Experimental Conditions.

The details of the experimental setup follow previous reports from our group (63, 64). In brief, two-dimensional electronic spectra were recorded using a diffractive optics-based, all-reflective 2D spectrometer providing phase stability of λ/160. Excitation pulses were generated by a home-built nonlinear optical parametric amplifier (NOPA), pumped by a commercial femtosecond laser system (Spectra Physics, Newport). The pulses were compressed to ∼12 fs using a combination of a deformable mirror (OKO Technologies, 19-channel) and a fused silica prism pair. Their temporal profile was characterized by frequency-resolved optical gating (FROG), with the traces analyzed using the commercial package FROG3 (Femtosecond Technologies). The resulting broadband spectrum had a ∼100 nm full width at half maximum (FWHM), centered at 620 nm, sufficient to cover the main electronic transitions into the lowest excited states. For 2DES measurements, three beams were focused onto the sample with a spot size of ∼130 μm, generating a photon-echo signal along the phase-matching direction. The emitted signal was collected using a Sciencetech 9055F spectrometer coupled to a CCD linear array detector (Entwicklungsbüro Stresing). For all experiments, the excitation pulse energy was attenuated to ∼10 nJ at a 1 kHz repetition rate. Phasing of the TG data was achieved using the “invariant theorem” procedure described in ref. 65.

### Theoretical Calculations.

We employed the ab-initio method to optimize the molecular structure of PSI complex. We then employed the TDDFT method to calculate the electronic ground and excited states. Before that, the molecular structures of subunits were also examined by frequency calculations at ground state, which allowed the molecular structure of pigments and associated protein matrix at their stable structure. Moreover, the excitonic couplings between pigments inside antenna complexes and also in core complexes have been investigated by dipole–dipole interactions. The electronic excited structures of pigments have been modeled as two-level model: electronic ground and excited states. The system Hamiltonian can be constructed with the optimal site energies and the off-diagonal elements, excitonic couplings. With these parameters, the system Hamiltonian can be written as

[1] $$\begin{array}{c} H_{S}=\sum_{i=1}^{N}|e_{i}\rangle \varepsilon_{i}\langle e_{i}|+\sum_{i=1}^{N}\sum_{i\neq j}|e_{i}\rangle V_{\mathrm{ij}}\left\langle e_{j}\right|, \end{array}$$

where $\varepsilon_{i}$ is the site energy (energy gap between ground and first electronic excited state) of i-th pigment. $V_{\mathrm{ij}}$ is the excitonic coupling between i- and j-th pigments. In PSI complex, we have sorted more than 133 pigments in 11 complexes. Thus, we have N=133. We also employed the system-bath model for the calculations of energy transfer. For this, the noisy environment can be modeled as infinity number of harmonic oscillators, thus, s we have

[2] $$\begin{array}{c} H_{\mathrm{env}}=\sum_{i=1}^{N}\sum_{k=1}^{N_{b}^{i}}\left(\frac{p_{\mathrm{ik}}^{2}}{2}+\frac{1}{2}\omega_{\mathrm{ik}}x_{\mathrm{ik}}^{2}\right). \end{array}$$

Here, ${\text{N}}_{b}^{i}$ is the number of bath modes coupled to pigment i, while x_*ik*_ and p_*ik*_ are the mass-weighted position and momentum of the k-th harmonic oscillator mode with frequency $\omega_{\mathrm{ik}}$. The system-bath coupling can be modeled as the interactions of electronic states to the coordinates of harmonic oscillators, which yields

[3] $$\begin{array}{c} H_{\mathrm{sb}}=\sum_{i}|e_{i}\rangle \langle e_{i}|\sum_{k}c_{\mathrm{ik}}x_{\mathrm{ik}}, \end{array}$$

where c_*ik*_ represents the coupling between the i-th pigment and the k-th pigment. The bath spectral density is defined as

[4] $$\begin{array}{c} J_{i}\left(\omega \right)=\pi \sum_{j}\frac{c_{\mathrm{ij}}^{2}}{2m_{\mathrm{ij}}\omega_{\mathrm{ij}}}\delta \left(\omega -\omega_{\mathrm{ij}}\right)=\gamma_{0}\omega e^{-\omega /\omega_{c}}, \end{array}$$

Here, $\gamma_{0}$ is the coupling strength, $\omega_{c}$ is the cut-off frequency of the bath. The influence of the bath can be fully described by its bath spectral density, which takes the form shown in Eq. **4** with parameters $\gamma_{0}=0.7$ cm^−1^ and $\omega_{c}=40$ cm^−1^. Moreover, the obtained site energies of pigments and the associated excitonic couplings between them have been described in *SI Appendix*.

In addition to the excitonic description, we further refined the Hamiltonian to account for low-energy states arising from environmental and electronic effects. The site energies obtained from ZINDO/S calculations incorporate pigment-specific electrostatic environments, enabling identification of red-shifted chlorophylls associated with both core and antenna complexes. To further capture the nature of long-wavelength states, charge-transfer (CT) contributions were included by introducing additional states coupled to the excitonic manifold. These CT states, which are known to contribute to red-shifted absorption in PSI systems, were incorporated phenomenologically into the Hamiltonian, allowing for mixed excitonic-CT character in the low-energy region. The resulting energy distribution and spatial localization of the red states are provided in *SI Appendix*, Table I and Fig. S5, where both their spectral positions and structural assignments are explicitly reported. This combined treatment enables a more comprehensive representation of the low-energy excitonic landscape relevant to energy transfer and trapping. The population dynamics has been calculated by the TNL method, the detailed description of it has been written in *SI Appendix*. With the optimal parameters and also the calculations, we obtained the time resolved population dynamics.

## Supplementary Material

Appendix 01 (PDF)

Dataset S01 (XLSX)

## Data Availability

The resulting matrix of the model Hamiltonian is provided in Dataset S1. All other study data are included in the article and/or supporting information.
