# Supplementary material for: Excitonic energy transfer in red algal Photosystem I reveals an evolutionary bridge between cyanobacteria and plants
Source: Proc Natl Acad Sci U S A. 2026 Jul 21;123(30):e2530661123. doi: 10.1073/pnas.2530661123 (PMC13416822; doi:10.1073/pnas.2530661123)
Supplement: Supplementary file 1 — Appendix 01 (PDF) [file pnas.2530661123.sapp.pdf]

Supplementary Information

Excitonic Energy Transfer in Red Algal Photosystem I Reveals an Evolutionary Bridge between Cyanobacteria and Plants

Mengyuan Cui[1,*], Zihui Liu[1,*], Miriam Izzo[2,*], Junhua Zhou[1,*], Enhu He[1], Vandana Tiwari[3,4], Petar H. Lambrev[5], R. J. Dwayne Miller[6], Joanna Kargul[2], Fulu Zheng[1,7], Ajay Jha[8,9], Hong-Guang Duan[1]

[1]*Department of Physics, School of Physical Science and Technology, Ningbo University, Ningbo, 315211, P.R. China*

[2]*Solar Fuels Laboratory, Center of New Technologies, University of Warsaw, 02-097, Warsaw, Poland*

[3]*Stanford PULSE Institute, SLAC National Accelerator Laboratory, Menlo Park, California 94025, United States*

[4]*Department of Chemical Science, Linac Coherent Light Source, SLAC National Accelerator Laboratory, Menlo Park, California 94025, United States*

[5]*Biological Research Centre, Szeged, Szeged 6726, Hungary*

[6]*Departments of Chemistry and Physics, University of Toronto, 80 St George Street, Toronto, M5S 3H6, Ontario, Canada.*

[7]*Zhejiang Key Laboratory of Advanced Optical Functional Materials and Devices, Ningbo University, Ningbo, 315211, China*

[8]*Rosalind Franklin Institute, Harwell, Oxfordshire OX11 0QX, United Kingdom*

[9]*Department of Pharmacology, University of Oxford, Oxford, OX1 3QT United Kingdom*

This supplementary information describes the theoretical framework employed, including the details of the model Hamiltonian, the system-bath model, and the *ab-initio* computational methodologies utilized for determining site energies and excitonic coupling parameters. Furthermore, we introduce the the red states of the PSI-LHCR complex and Time-Nonlocal (TNL) quantum master equation, global fitting approach and details of the data treatment procedures, including detailed analyses of residuals. Finally, the supplementary material also introduces the 2DES and resulted data of PSI complex at different temperatures.

I. SAMPLE PREPARATION AND PURIFICATION

A. Culturing of His$_6$-PsaD-PSI *C. merolae* cells and isolation of thylakoid membranes

The genetic strategy to introduce the modified His$_6$-tagged PsaD construct (CMV144CT, 417 bp) into the URA locus of the *Cyanidioschyzon merolae* M4 mutant was adapted from the protocol of Fujiwara *et al.* [1]. The biochemical characterization of the recombinant PSI complex for assessment of purity, structural integrity, and photochemical activity of the biophotocatalyst, in comparison with the native PSI complex, are reported in Izzo *et al.* [2]. The engineered *C. merolae* strain was cultivated in Allen 2 medium (pH 2.5) at 42 $°C$ under continuous white light illumination (90 μmol photons$\cdot$m$^{-2}\cdot$s^{-1}; Panasonic FL40SS-ENW/37) with gentle bubbling with 3-5% CO_2 in air, as previously described in [3] and [4]. Cultures (9 L) were grown to an OD_{680} of 0.9-1.0 for isolation of thylakoids and PSI purification, as described in Haniewicz *et al.* [4]. For thylakoids isolation, cells were disrupted on ice with 0.1 mm glass beads for 13 cycles (10 s 'on', 4 min 'off') in buffer A (10 mM $CaCl_2$, 5 mM $MgCl_2$, 25% (w/v) glycerol, 40 mM MES-NaOH, pH 6.1) supplemented with DNase I (5 mg), RNase (10 µL), and a protease inhibitor cocktail (Thermo Fisher) (1 tablet per 50 mL). The homogenate was filtered (Whatman paper), then centrifuged at 180,000×g for 25 min at 4 $°C$ to pellet thylakoids. Thylakoid membranes were washed three times with buffer A, resuspended to a final Chl a concentration of 2-5 mg$\cdot$mL^{-1}, then snap-frozen in liquid nitrogen for further use.

B. Purification of His-tagged PSI Complex and Biochemical Characterization

The His_6-PsaD-PSI complex was purified, as reported in Izzo *et al* [2]. Briefly, the His-tagged complex was purified using immobilized metal affinity chromatography (IMAC). Thylakoids (1 mg Chl a mL^{-1}) were solubilized with 1% (w/v) n-dodecyl-β-D-maltoside (DDM) in the dark on ice for 40 min. All the subsequent procedures were performed at 4°C. The solubilized fraction was filtered and loaded onto a 1 mL HisTrap^{TM} HP column (Cytiva), pre-equilibrated with 5 column volumes (CV) of the wash buffer (3 mM $CaCl_2$, 0.03% DDM, 25% glycerol, 20 mM imidazole, 40 mM HEPES-NaOH, pH 8.0). After washing with 10 CV of the wash buffer, bound PSI complex was eluted using a linear imidazole gradient (0-1 M) in the same buffer. Eluted fractions were analyzed via UV-VIS absorbance spectroscopy (Shimadzu UV-VIS 1800) (Fig. S1A. Imidazole was removed by buffer exchange using Vivaspin-20 concentrators (100 kDa MWCO, Sartorius) at 4000×g, 4 for 15 min. Elution buffer was diluted 1:1 with wash buffer without imidazole and the exchange repeated two more times.

The His_6-PsaD-PSI complex was concentrated to 2-5 mg Chl a mL^{-1} and stored in aliquots at -80^{circ}C until further use. Protein purity was assessed by SDS-PAGE on 14-20% acrylamide gradient gels (with 0.2-0.4% bis-acrylamide, 12-17% sucrose, 0.1% SDS, and 0.42 M Tris-HCl, pH 9.2), using a 6% stacking gel (0.16% bis-acrylamide, 0.1% SDS, 54 mM Tris-HCl, pH 6.1). Samples were prepared in a Laemmli buffer (Roti Load 1, Roth GmbH), and loaded at 1 μg (thylakoids) or 2.5 μg (PSI) of Chl a per lane. Electrophoresis was run using a Hoefer SE 400 system (see Fig. S1B).

Photochemical activity of PSI samples was measured via the oxygen consumption assay using a Clark-type electrode (Hansatech), as described in Haniewicz *et al.* [4]. Measurements (1 μg Chl a per measurement) were performed at 30 ^{circ}C in 40 mM HEPES-NaOH (pH 8.0), supplemented with 3 mM $CaCl_2$, 0.03% DDM, and 25% glycerol. Additives included 0.2 mM methyl viologen (artificial electron acceptor), 0.2 mM dichlorophenolindophenol (artificial electron mediator), and 10 mM sodium azide. Following 2 min of dark adaptation, 0.6 mM sodium ascorbate (electron donor) was added, and the reaction mixture was illuminated with 5000 µmol photons·m^{-2}·s^{-1} using a KL 2500 LCD lightbox (Schott) with a cold white light source. Each measurement was performed in two replicates (n = 2) (See Fig. S2).

C. Biochemical characterization of the His6-PsaD-PSI complex

The biochemical characterization of the IMAC-purified His_6-PsaD-PSI complex was performed to confirm its purity and activity. To this end, UV-VIS spectrum of the freshly purified His_6-PsaD-PSI was recorded (Fig. S1A. The SDS-PAGE electrophoresis analysis was performed to confirm the homogeneity of the PSI sample (Fig. S1B, while the photochemical activity was assessed by measuring oxygen consumption (Fig. S2), as described in Materials and Methods.

II. MODEL HAMILTONIAN AND PARAMETERS

A. Hamiltonian Matrix of PSI complex

The PSI complex contains 133 spectroscopically relevant chlorophyll-a (Chl-a) molecules. In this study, we do not consider the spectroscopic signals from carotenoids. The determination of the 133-dimensional model Hamiltonian and its matrix elements was carried out as follows.The protein structure of the PSI complex was taken from the PDB file with accession code 7BLZ. The molecular structures of the pigments were extracted directly from the same PDB file and subsequently refined using density functional theory (DFT). The excitonic couplings between pigments were calculated using the transition density cube method; A detailed description of this method is provided in Ref. [5].The optimized pigment molecules were subjected to quantum chemical calculations using the ZINDO/S method[6], from which the low-lying excited states and their corresponding energies were obtained.Subsequently, the site energies were optimized by simultaneously fitting the resulting absorption spectra to available experimental data.The resulting matrix of the model Hamiltonian is listed in the attached Excel file.

B. Linear absorption and stick spectra

Here, we show the calculated stick spectrum of PSI complex(see Fig. S3), the values of site energies and excitonic couplings are taken from the *ab-initio* method. The details have been written in the last section. We show the calculated sticks and to fit with the measured absorption spectrum of PSI complex (red solid line) at room temperature.

C. The CT states of PSI-LHCR complex

In this section,to better account for the mixed excitonic–CT character in the low-energy manifold, we incorporated CT states into the Hamiltonian—states known to contribute to red-shifted absorption, particularly in PSI core complexes and higher plants. We calculated the population over 30 ps at temperatures of 300 K, 77 K, 40 K, and 20 K (see Fig. S4).In the Hamiltonian, we incorporate four CT states: $ec2A^+ec3A^-$, $ec2B^+ec3B^-$, $P700^+ec3A^-$, $P700^+ec3B^-$, and the coupling parameters between the CT states and the relevant exciton states are set to 100 cm^{-1}. The specific parameters among the CT states are listed in Tab. I.

III. RED STATES OF PSI-LHCR COMPLEX

To substantiate the assignment of red states, we analyzed the distribution of low-energy excitonic states across the PSI-LHCR complex by combining structure-based calculations with experimental validation. Red states were defined as chlorophylls exhibiting site energies corresponding to wavelengths longer than 685 nm, consistent with their role as low-energy exciton sinks. Based on this criterion, a total of 17 red chlorophylls were identified. These pigments are spatially distributed across both the LHCR antenna and the PSI core. Specifically, two chlorophylls are located in Lhcr1, one in Lhcr2, three in Lhcr3, four in PsaA, five in PsaB, one in PsaI, and one in PsaO. This distribution highlights the presence of a dual-sink architecture, with red states residing both near the reaction centre and within peripheral antenna complexes. Such localization supports their functional role in capturing long-wavelength photons and mediating energy transfer under varying energetic conditions. The site energies and excitonic states were obtained from structure-refined geometries (DFT) combined with ZINDO/S parametrization, and used to construct the excitonic Hamiltonian. The validity of this assignment was further confirmed by comparison between the calculated absorption spectrum, obtained using a time-nonlocal response formalism, and the experimental spectrum, showing excellent agreement. Importantly, the identified red-state energy range (703 nm and 685 - 688 nm) is consistent with previously reported long-wavelength chlorophyll forms in PSI. The spatial localization and spectral positions of these states are presented in Fig. S5, providing direct support for their assignment to both core and antenna subunits. While individual contributions from specific red states may be partially obscured due to line broadening arising from inhomogeneous disorder and ultrafast decoherence, their cumulative effect is manifested as a distinct red-shift in the overall absorption profile. This behavior is consistent with the presence of distributed low-energy excitonic states across the PSI-LHCR complex.

IV. GLOBAL FITTING APPROACH AND THE DECAY ASSOCIATED SPECTRA

This section outlines the multidimensional global fitting approach employed for analyzing experimental 2D spectral arrays. The procedure follows established algorithms [7], wherein a sequence of 2D spectra acquired at distinct waiting times T is compiled into a three-dimensional data set S(ω_τ, ω_t, T). This data set undergoes decomposition into a superposition of two-dimensional decay-associated spectra (2D-DAS) $A_i(\omega_\tau, \omega_t)$, each characterized by an exponential decay with lifetime τ_i:

$$S(\omega_\tau, \omega_t, T) = \sum_{i=1}^{N} A_i(\omega_\tau, \omega_t) \exp(-T/\tau_i). \tag{S1}$$

The 2D-DAS amplitudes encode critical dynamical information: positive (red) amplitudes signify exponential decay components with lifetime τ_i, while, the negative (blue) amplitudes indicate exponential rising components governed by the same τ_i. To minimize the influence of pulse overlap, the fitting procedure begins at a waiting time of 45 fs. The temporal measurement window spans up to 2.0 ps with a time step of 15 fs. Given the presence of multi-scale dynamics, the kinetics are modeled using a biexponential function, $N = 5$. This approach reveals evolving spectral features beyond the capabilities of transient absorption (TA) spectroscopy, providing resolved spatio-temporal correlations across the (ω_τ, ω_t) frequency domain.

V. TIME NON-LOCAL QUANTUM MASTER EQUATION

We used the time non-local method for the simulation of this paper [8, 9]. ρ is the density matrix of the system and bath. Its time evolution is given by the Liouville-von Neumann equation with the Liouville superoperator $\mathcal{L}$ ($\hbar = 1$),

according to

$$\dot{\rho} = -i[H_{\rm tot}, \rho] = \mathcal{L}\rho. \tag{S2}$$

The total Hamiltonian $H_{\rm tot} = H_{\rm s} + H_{\rm b} + \lambda H_{\rm sb} + \lambda^2 H_{\rm ren}$ comprises the system, bath, interaction and renormalization terms. For a single system with degree of freedom x, the bath consists of an ensemble of harmonic oscillators, i.e, $H_{\rm b} = \sum_{j=1}^{N} \left[p_j^2/(2m_j) + m_j\omega_j^2 x_j^2/2\right]$ and the standard form of the coupling is $H_{\rm sb} = f(x)\sum_{j=1}^{N} c_j x_j$ with some real function $f(\cdot)$.

The projection scheme of Nakajima and Zwanzig [10] can separating the dynamics of the bath the system. The thermal state of the bath is described by the canonical density $\rho_{\rm b}^{\rm eq} = \exp(-\beta H_{\rm b})$ with temperature $T = (k_B\beta)^{-1}$. Applying the projector $P = \rho_{\rm b}^{\rm eq}{\rm tr}_{\rm b}$, with ${\rm tr}_{\rm b}\rho_{\rm b}^{\rm eq} = 1$ and $Q = (1-P)$ yields the exact formal quantum master equation for the time evolution of the reduced system density operator $\rho_{\rm s}$ [8] in the form of

$$\begin{aligned}
\dot{\rho}_{\rm s}(t) &= \mathcal{L}_{\rm s}^{\rm eff}\rho_{\rm s}(t) + \int_0^t K(t,t')\rho_{\rm s}(t') + \Gamma(t),\\
\mathcal{L}_{\rm s}^{\rm eff} &= \mathcal{L}_{\rm s} + \lambda\,{\rm tr}_{\rm b}\mathcal{L}_{\rm sb}\rho_{\rm b}^{\rm eq} + \lambda^2\mathcal{L}_{\rm ren},\\
K(t,t') &= \lambda\,{\rm tr}_{\rm b}\mathcal{L}_{\rm sb}\left(\mathcal{T}e^{\int_{t'}^{t} Q\mathcal{L}dt''}\right)Q(\mathcal{L}_{\rm b} + \lambda\mathcal{L}_{\rm sb})\rho_{\rm b}^{\rm eq},\\
\Gamma(t) &= \lambda\,{\rm tr}_{\rm b}\mathcal{L}_{\rm sb}\left(\mathcal{T}e^{\int_{0}^{t} Q\mathcal{L}dt''}\right)Q\rho_{\rm tot}(0).
\end{aligned} \tag{S3}$$

Here, $\rho_{\rm tot}(0)$ is the total density operator of system and bath at initial time. Moreover, we use $\mathcal{L}_{\rm s}$, $\mathcal{L}_{\rm sb}$, and $\mathcal{L}_{\rm ren}$ for the corresponding parts of the Liouville superoperator which are associated to the respective Hamilton operators. Moreover, $\mathcal{L}_{\rm s}^{\rm eff} = -i[H_{\rm s} + H_{\rm ren}, \cdot]$ and $\mathcal{T}$ is the time-ordering operator [8]. Next, we expand the correlated thermal equilibrium state to first order in the overall coupling strength λ and obtain

$$\rho^{\rm eq} \approx \frac{1}{Z_{\rm s}}\frac{1}{Z_{\rm b}}e^{-\beta(H_{\rm s}+H_{\rm b})} - \lambda\frac{1}{Z_{\rm s}}\frac{1}{Z_{\rm b}}\int_0^\beta d\beta' e^{-(\beta-\beta')(H_{\rm s}+H_{\rm b})H_{\rm sb}^{(1)}e^{-\beta'(H_{\rm s}+H_{\rm b})}}, \tag{S4}$$

with the respective partition functions $Z_{\rm tot} = {\rm tr}\exp(-\beta H_{\rm tot})$, $Z_{\rm b} = {\rm tr}_{\rm b}\exp(-\beta H_{\rm b})$ and $Z_{\rm s} = {\rm tr}_{\rm s}\exp(-\beta H_{\rm s})$. Next, we take the trace over the system degrees of freedom on both sides of Eq. (S4) and get

$$\rho_{\rm b}^{\rm eq} = \frac{1}{Z_{\rm b}}e^{-\beta H_{\rm b}} + \frac{\lambda\chi}{Z_{\rm b}}\int_0^\beta e^{-(\beta-\beta')H_{\rm b}}\left(\sum_{i=1}^{N} c_i x_i\right)e^{-\beta' H_{\rm b}}. \tag{S5}$$

Here, $\chi = (1/Z_{\rm s}){\rm tr}_{\rm s}\left[f(x)e^{-\beta H_{\rm s}}\right]$. The well-known bath correlation function

$$c(t) = \int_{-\infty}^{\infty}\frac{d\omega}{2\pi}J(\omega)\cos(\omega t)\coth\left(\frac{\beta\omega}{2}\right) - i\int_{-\infty}^{\infty}\frac{d\omega}{2\pi}J(\omega)\sin(\omega t) \equiv a(t) - ib(t) \tag{S6}$$

is given in terms of the standard bath spectral density $J(\omega)$ and has the real part $a(t)$ and imaginary part $b(t)$. Inserting Eqs. (S4) and (S5) into Eq. (S3), we can express the last three terms of Eq. (S3) by $a(t)$ and $b(t)$ according to

$$\begin{aligned}
\mathcal{L}_{\rm s}^{\rm eff} &= \mathcal{L}_{\rm s} + \lambda^2\mu\mathcal{L}_{\rm ren,s} + \lambda^2\chi\mu\mathcal{L}^-,\\
K(t,t') &= \lambda^2\mathcal{L}^-\left(a(t-t')\mathcal{T}e^{\int_{t'}^{t}\mathcal{L}_{\rm s}}\mathcal{L}^- + b(t-t')\mathcal{T}e^{\int_{t'}^{t}\mathcal{L}_{\rm s}}\mathcal{L}^+\right),\\
\Gamma(t) &= \lambda^2\mathcal{L}^-\int_{-\infty}^{0}dt'\left[a(t-t')\mathcal{T}e^{\int_{t'}^{t}\mathcal{L}_{\rm s}}\mathcal{L}^-\rho_{\rm s}^{\rm eq} + b(t-t')\mathcal{T}e^{\int_{t'}^{t}\mathcal{L}_{\rm s}}\mathcal{L}^+\rho_{\rm s}^{\rm eq}\right],
\end{aligned} \tag{S7}$$

with $\mathcal{L}^- = -i[H_{\rm sb}, \cdot]$ and $\mathcal{L}^+ = [H_{\rm sb}, \cdot]_+ - 2\chi$. In terms of the spectral density, the potential renormalization is given by $\mu = \int_{-\infty}^{\infty}\frac{d\omega}{2\pi}J(\omega)/\omega$.

In order to obtain an analytic form of the bath correlation function, any given spectral density (in our particular case, we use the standard Ohmic form) can be approximated by a sum of Lorentzian-like spectral terms [8, 9] according to

$$J(\omega) = \frac{\pi}{2}\sum_{k=1}^{n}\frac{p_k\omega}{[(\omega+\Omega_k)^2 + \Gamma_k^2][(\omega-\Omega_k)^2 + \Gamma_k^2]}, \tag{S8}$$

where the spectral amplitude p_k, the frequency Ω_k and the width Γ_k following from the expansion of the original function in terms of the Lorentzian-like terms. Using the expanded form in Eq. (S6), we get

$$\begin{aligned} a(t) &= \sum_{k=1}^{n} \frac{p_k}{8\Omega_k\Gamma_k} \coth\left[\frac{\beta}{2}\left(\Omega_k + i\Gamma_k\right) e^{i\Omega_k t - \Gamma_k t}\right] + \sum_{k=1}^{n} \frac{p_k}{8\Omega_k\Gamma_k} \coth\left[\frac{\beta}{2}\left(\Omega_k - i\Gamma_k\right) e^{-i\Omega_k t - \Gamma_k t}\right] + \frac{2i}{\beta}\sum_{k=1}^{n'} J(i\nu_k) e^{-\nu_k t}, \\ b(t) &= \sum_{k=1}^{n} \frac{i p_k}{8\Omega_k\Gamma_k}\left(e^{i\Omega_k t - \Gamma_k t} - e^{-i\Omega_k t - \Gamma_k t}\right), \end{aligned} \tag{S9}$$

with the Matsubara frequencies $\nu_k = 2\pi k/\beta$.

Next, we rewrite the correlation functions as $a(t) = \sum_{k=1}^{n_r} \alpha_k^r e^{\gamma_k^r t}$ and $b(t) = \sum_{k=1}^{n_i} \alpha_k^i e^{\gamma_k^i t}$ with $n_i = 2n$, $n_r = 2n+n'$, where n' is the number of Matsubara frequencies used. Then, we define new auxiliary "density matrices" which incorporate both memory effects and initial correlations according to

$$\begin{aligned} \rho_k^r(t) &= \lambda\left(\mathcal{T} e^{\int_0^t dt' \mathcal{L}_s} e^{\gamma_k^r t} \int_0^\infty dt' e^{\mathcal{L}_s t'} e^{\gamma_k^r t'} \mathcal{L}^- \rho_s^{eq} + \int_0^t dt' e^{\gamma_k^r (t-t')} \mathcal{T} e^{\int_{t'}^t dt' \mathcal{L}_s} \mathcal{L}^- \rho_s(t')\right), \\ \rho_k^i(t) &= \lambda\left(\mathcal{T} e^{\int_0^t dt' \mathcal{L}_s} e^{\gamma_k^i t} \int_0^\infty dt' e^{\mathcal{L}_s t'} e^{\gamma_k^i t'} \mathcal{L}^+ \rho_s^{eq} + \int_0^t dt' e^{\gamma_k^i (t-t')} \mathcal{T} e^{\int_{t'}^t dt' \mathcal{L}_s} \mathcal{L}^+ \rho_s(t')\right). \end{aligned} \tag{S10}$$

The time-retarded Eq. (S3) (first term) can then be deconvoluted into the coupled first-order equations

$$\begin{aligned} \dot\rho_s(t) &= \mathcal{L}_s^{eff}(t)\rho_s(t) + \lambda\left[\sum_{k=1}^{n_r} \alpha_k^r \mathcal{L}^- \rho_k^r(t) + \sum_{k=1}^{n_i} \alpha_k^i \mathcal{L}^- \rho_k^i(t)\right], \\ \dot\rho_k^r(t) &= (\mathcal{L}_s(t) + \gamma_k^r)\rho_k^r(t) + \lambda\mathcal{L}^-\rho_s(t), k = 1, \ldots, n_r, \\ \dot\rho_k^i(t) &= (\mathcal{L}_s(t) + \gamma_k^i)\rho_k^i(t) + \lambda\mathcal{L}^+\rho_s(t), k = 1, \ldots, n_i. \end{aligned} \tag{S11}$$

This set of time non-local quantum master equations was used to calculate quantum system dynamics.

VI. TWO-DIMENSIONAL VIBRATIONAL MAPS

In this section, we outline the procedure used to generate 2D vibrational maps. After applying the global fitting protocol, the resulting three-dimensional residuals were subjected to Fourier transformation along the waiting time axis T. This isolates oscillatory contributions and projects them onto both excitation and detection frequency axes, yielding 2D vibrational maps that identify the spectral locations of coherent amplitudes. For each vibrational frequency ω_T, we extracted both the peak amplitude and integrated intensity, while dashed lines were added to indicate the expected vibrational progressions of the relevant modes.

To minimize artifacts from pulse overlap, the fitting was initiated at 45 fs. Measurements were recorded over a time window of 2.0 ps with 15 fs increments. Reliable fits required at least a biexponential function to capture the kinetics in the 3D dataset. Residuals were then obtained by subtracting these kinetic components, and Fourier transformation of the residuals along T produced the frequency-resolved maps. This approach provided a frequency resolution of approximately 16.5 cm^{-1} in the 2D vibrational spectra.The 2D vibrational maps at 8 K in Fig. S6.

VII. MEASURED 2DES OF PSI COMPLEX AT 80K

In this section, we show the measured 2D electronic spectra of the PSI complex at 80 K. In Fig. S7, the real part of the 2D electronic spectra have been shown with selected waiting time 80, 150, 300, 600, 1094 and 1965 fs. The positive and negative magnitudes in the 2D spectra indicate the transitions associated with the ground-state bleach (GSB) and the excited-state absorption (ESA), respectively. Moreover, the selected peaks are marked as g, h, i and j in Fig. S7D. The associated traces are plotted as red solid lines in Fig. S7G-J, respectively. The curves are fitted by exponential functions, which has been shown as black dashed lines accordingly.

The resulted data of 2DES with different waiting times are collected to analyze their decay dynamics. For this, we employed the global fitting approach to treat the data and we obtained 5 components with decay-associated spectra. The resulted data are shown in Fig. S8. The reported data shows the timescales of 0.8 ps for the fastest component. They show the red peak centered at around (ω_t, ω_τ) = (14700, 14600) cm^{-1}, which indicates a fast decay in that

frequency region. Moreover, we also plotted the second component with decay timescale of 4 ps, which show a strong red peak centered at the similar region. In addition, the decay associated spectra with timescales of 18 ps, 537 ps and Inf components are shown as well. To disentangle the origins of these peaks, we also plotted the contour lines of 2DES.

Moreover, we also performed the data analysis of the 2D vibrational maps for the PSI complex, which measured at 80 K. The 2D residuals are collected with different waiting times and 3D data was obtained. We then performed the Fourier transform of the 2D residuals to get the 2D vibrational maps, which has been plotted in Fig. S9.

VIII. 2DES WITH LONG DETECTION TIME WINDOW

In this section, we show the measured 2DES with long detection window. The measured 2DES of 80 K and 8 K are shown in Fig. S10 and Fig. S11.

IX. EXTRACTED TIMESCALES OF EXCITONIC ENERGY TRANSFER

In this section, we show the timescales retrieved from population dynamics obtained from experiments at different temperatures and theoretical calculations on the PSI complex. The values obtained from theoretical calculations are listed in Tab. II, and those obtained at 80 K and 8 K are listed in Tab. III. The unit is defined as femtosecond.

[1] Fujiwara, T. *et al.*, Gene Targeting in the Red Alga Cyanidioschyzon merolae: Single- and Multi-Copy Insertion Using Authentic and Chimeric Selection Markers. PLoS ONE, **8** e73608 (2013).

[2] Izzo, M. *et al.*, Development of a Novel Nanoarchitecture of the Robust Photosystem I from a Volcanic Microalga Cyanidioschyzon merolae on Single Layer Graphene for Improved Photocurrent Generation, Int. J. Mol. Sci., **22**, 16, (2021).

[3] Krupnik, T. *et al.*, A Reaction Center-dependent Photoprotection Mechanism in a Highly Robust Photosystem II from an Extremophilic Red Alga, Cyanidioschyzon merolae. J. Biol. Chem. **288**, 23529 (2013).

[4] Haniewicz, P. *et al.*, Molecular Mechanisms of Photoadaptation of Photosystem I Supercomplex from an Evolutionary Cyanobacterial/Algal Intermediate. Plant Physiol. **176**, 1433 (2018).

[5] Fr"ahmcke, J. S. *et al.*, Coulombic Couplings Between Pigments in the Major Light-Harvesting Complex LHC II Calculated by the Transition Density Cube Method. Chem. Phys. Lett. **430**, 397 (2006).

[6] Frisch, M. J.; Trucks, G. W.; Schlegel, H. B.; Scuseria, G. E.; Robb, M. A.; Cheeseman, J. R.; Scalmani, G.; Barone, V.; Mennucci, B.; Petersson, G. A.; Nakatsuji, H.; Caricato, M.; Li, X.; Hratchian, H. P.; Izmaylov, A. F.; Bloino, J.; Zheng, G.; Sonnenberg, J. L.; Hada, M.; Ehara, M.; Toyota, K.; Fukuda, R.; Hasegawa, J.; Ishida, M.; Nakajima, T.; Honda, Y.; Kitao, O.; Nakai, H.; Vreven, T.; Montgomery, J. A., Jr.; Peralta, J. E.; Ogliaro, F.; Bearpark, M.; Heyd, J. J.; Brothers, E.; Kudin, K. N.; Staroverov, V. N.; Kobayashi, R.; Normand, J.; Raghavachari, K.; Rendell, A.; Burant, J. C.; Iyengar, S. S.; Tomasi, J.; Cossi, M.; Rega, N.; Millam, J. M.; Klene, M.; Knox, J. E.; Cross, J. B.; Bakken, V.; Adamo, C.; Jaramillo, J.; Gomperts, R.; Stratmann, R. E.; Yazyev, O.; Austin, A. J.; Cammi, R.; Pomelli, C.; Ochterski, J. W.; Martin, R. L.; Morokuma, K.; Zakrzewski, V. G.; Voth, G. A.; Salvador, P.; Dannenberg, J. J.; Dapprich, S.; Daniels, A. D.; Farkas .; Foresman, J. B.; Ortiz, J. V.; Cioslowski, J.; Fox, D. J. Gaussian, Inc., Wallingford CT, 2009.

[7] Prokhorenko, V. I. European Photochemistry Association Newletter June 2012, p21.

[8] C. Meier, and D. J. Tannor, J. Chem. Phys. **111**, 3365 (1999).

[9] U. Kleinekathöfer, J. Chem. Phys. **121**, 2505 (2004).

[10] R. Zwanzig, *Lectures in Theoretical Physics*, Boulder, Colorado (Interscience, New York, 1961), Vol. 3.

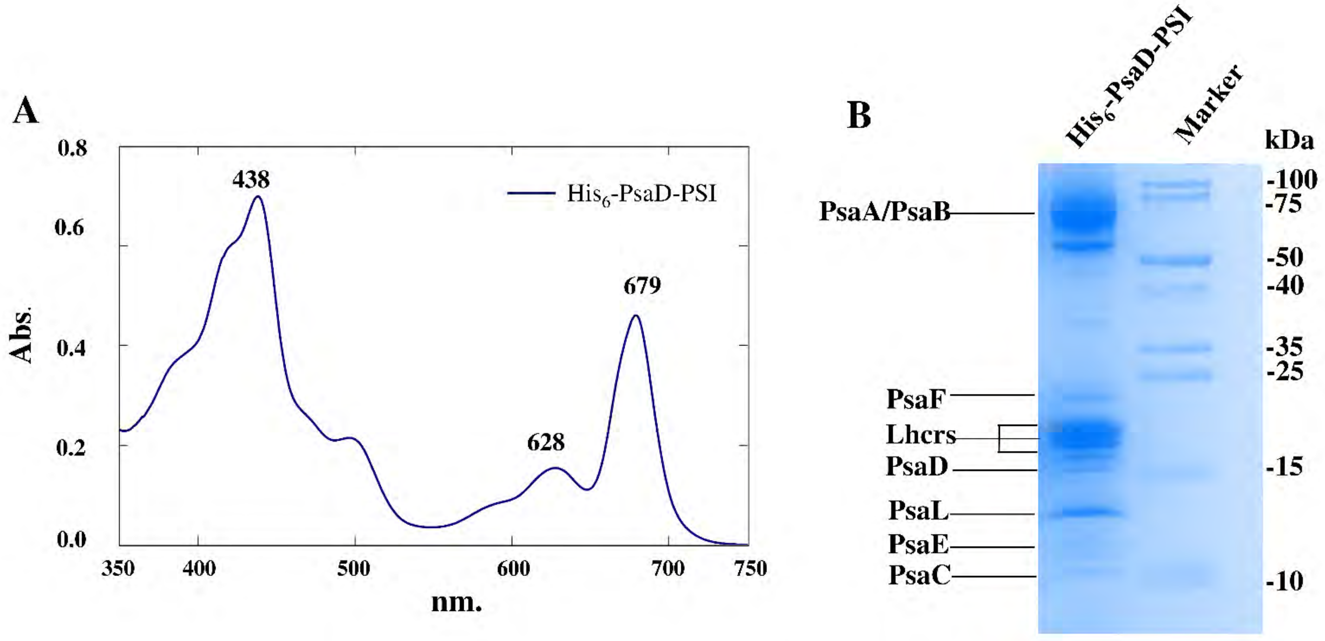

FIG. S1: Biochemical characterization of the His_6-PsaD-PSI complex from *C. merolae.* (A) RT absorption spectrum of His_6-PsaD-PSI complex (blue solid line). (B) SDS-PAGE analysis the purified His_6-PsaD-PSI complex (2 νg Chl a per lane). Marker, protein size marker.

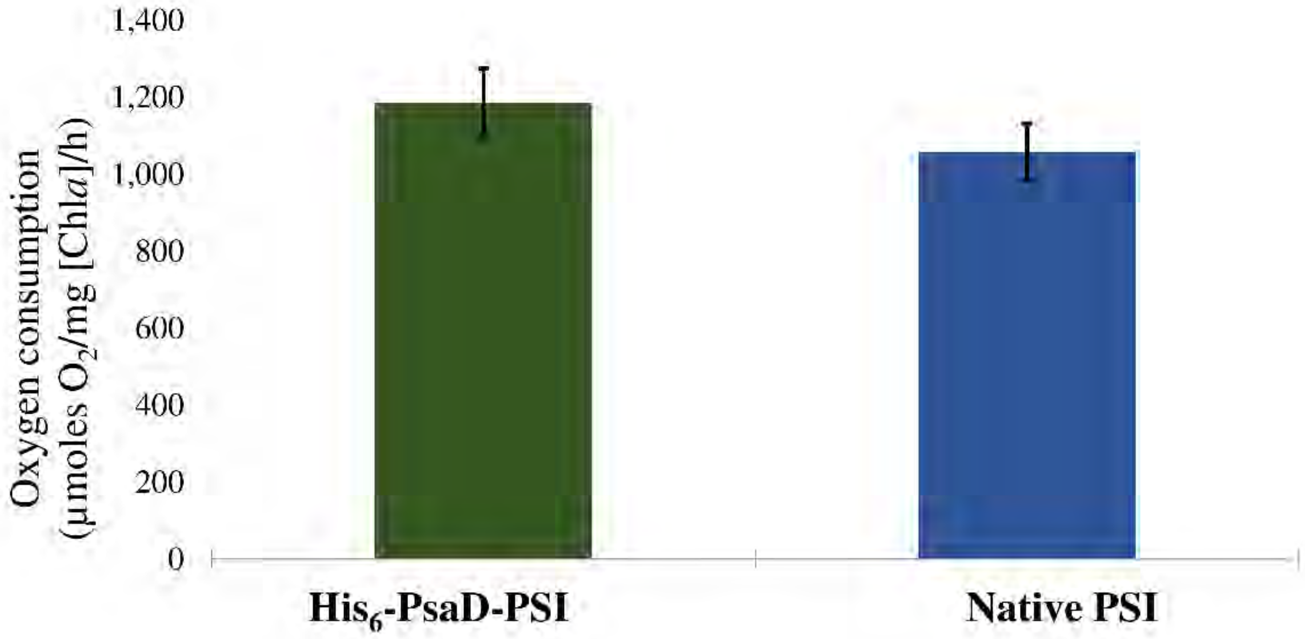

FIG. S2: Photochemical activity of the purified His$_6$-tagged *C. merolae* PSI complex. The values were obtained from two independent measurements (n = 2) and expressed as means ±SD.

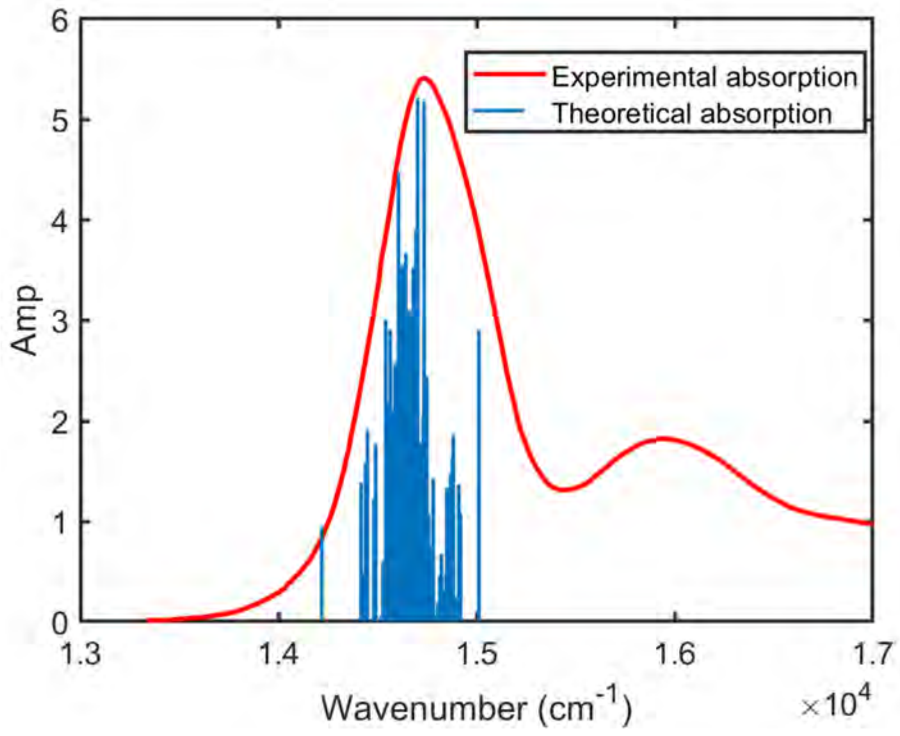

FIG. S3: Calculated sticks overlapped with the measured absorption (red solid line).

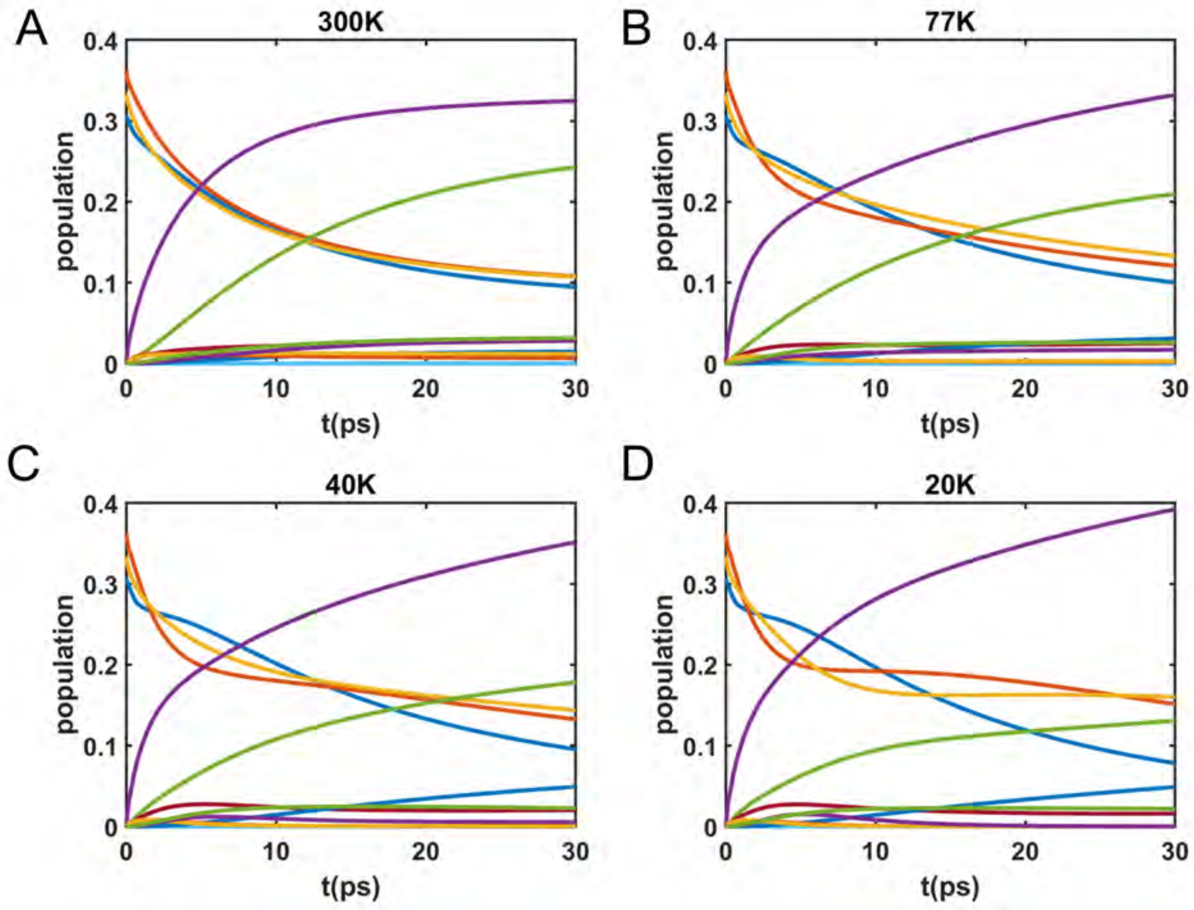

FIG. S4: The population over 30 ps at temperatures of 300 K, 77 K, 40 K, and 20 K

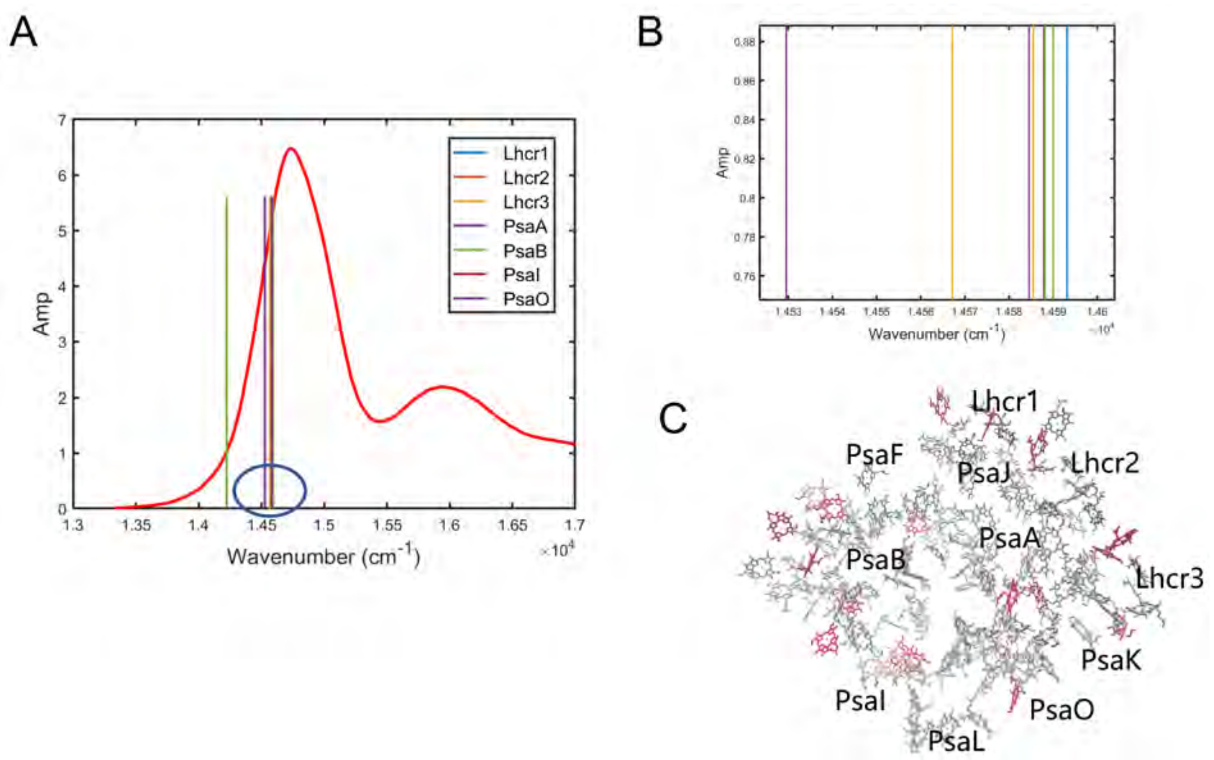

FIG. S5: (A) Experimental absorption spectrum of the PSI-LHCR complex (solid red line) overlaid with the calculated positions of red states derived from the excitonic Hamiltonian. Vertical lines indicate the energies of the identified low-energy (red) states associated with individual subunits (Lhcr1-3, PsaA, PsaB, PsaI, and PsaO), highlighting their spectral contributions to the low-energy manifold. (B) Expanded view of the low-energy spectral region, illustrating the distinct separation and clustering of calculated red-state energies. (C) Structural representation of the PSI-LHCR complex illustrating the spatial localization of the red states. Chlorophylls contributing to red-shifted excitonic states are highlighted (red) within both the core (PsaA/B and associated subunits) and LHCR antenna complexes, demonstrating the distributed nature of low-energy exciton sinks across the protein architecture.

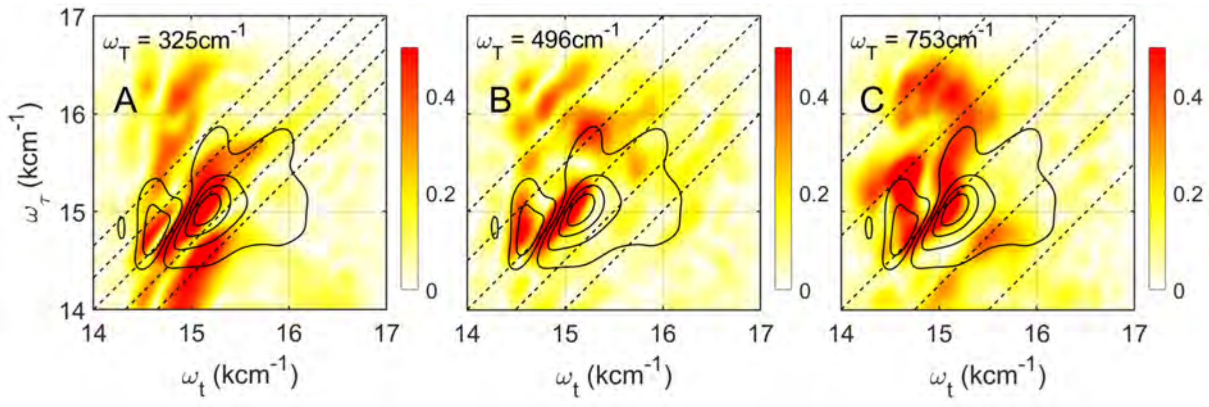

FIG. S6: (A-C) Fourier-transformed oscillatory maps reveal vibrational coherences with characteristic frequencies of 325, 496, and 753 cm^{-1} at 8 K.

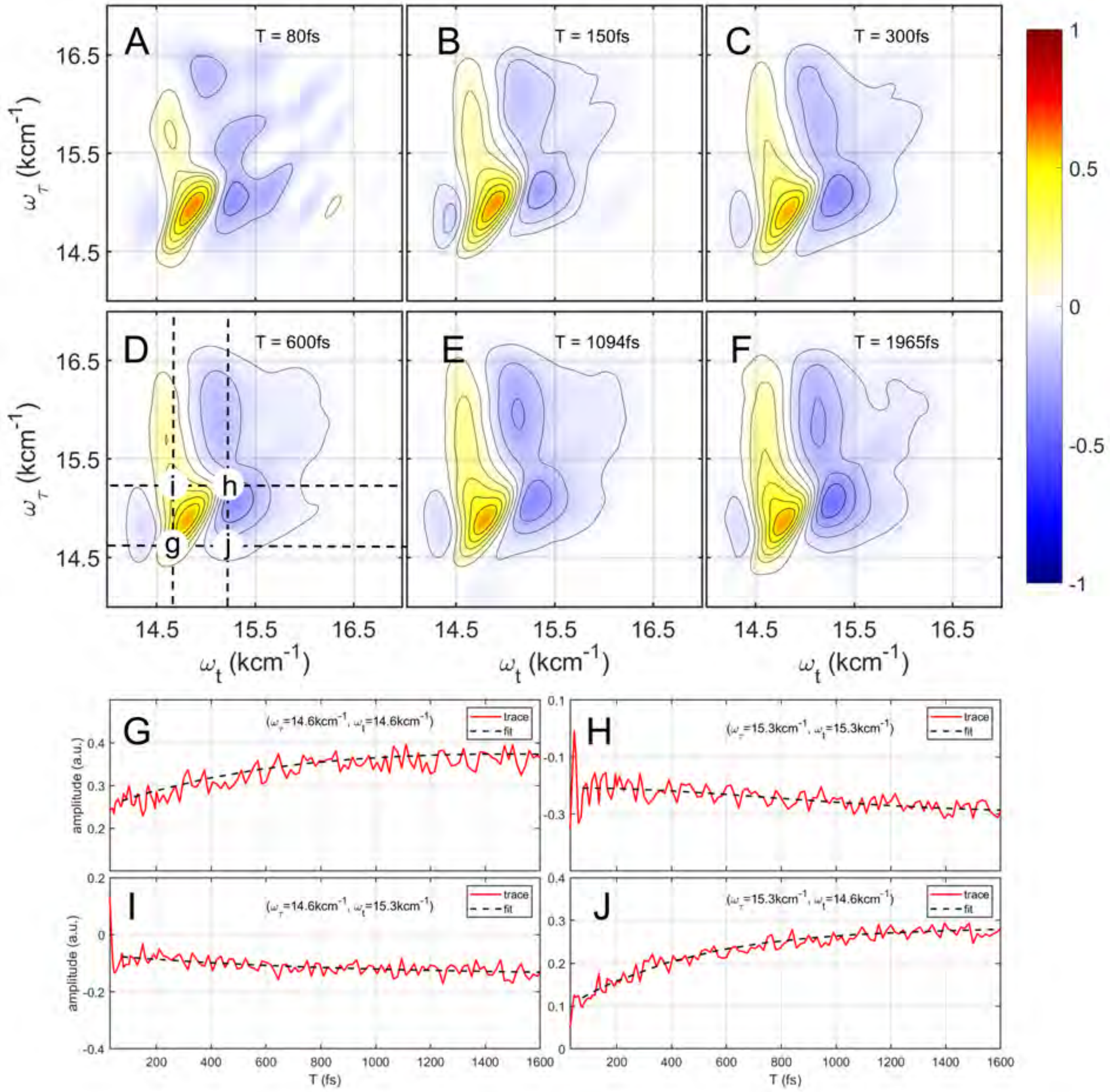

FIG. S7: Measured 2DES of PSI complex at 80 K with selected waiting times. The associated main and cross peaks are labeled in (D) with i, h, g, j. The time-resolved traces are plotted as red solid lines with curve fitting results (black dashed lines).

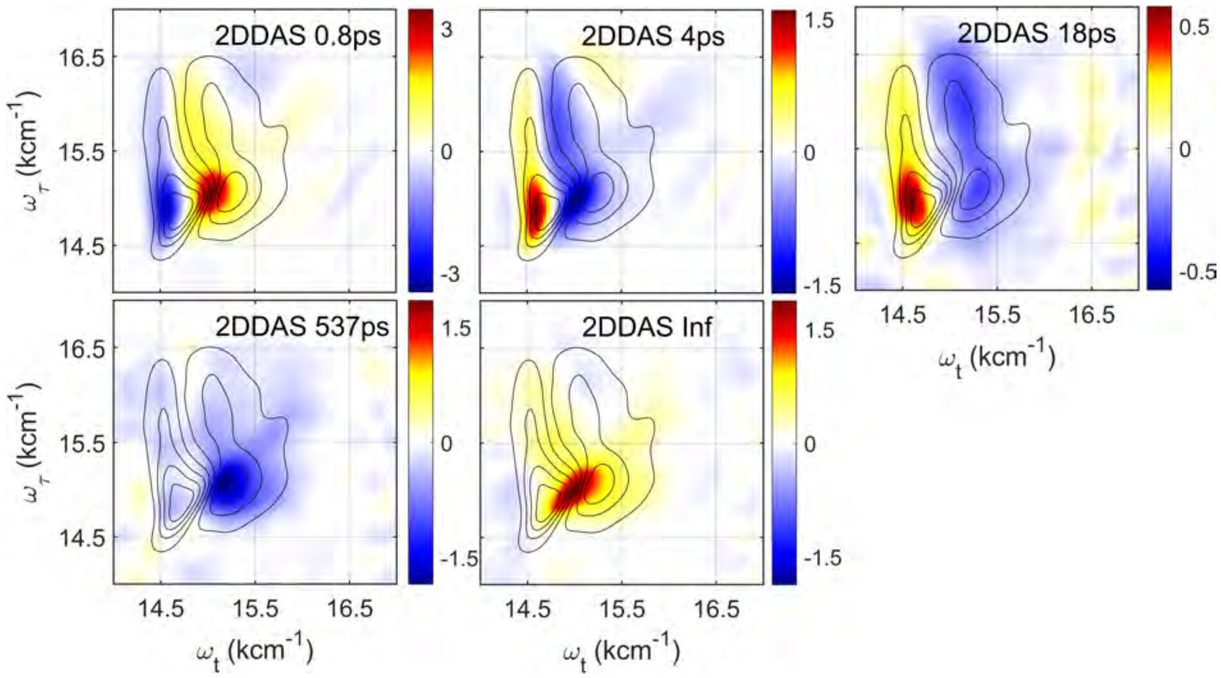

FIG. S8: The 2D-DAS with the corresponding resolved decay time constant. The fastest DAS component shows the lifetime of 0.8 ps. The other components belong to the timescale of 4 ps, 18 ps, 537 ps and infinity, respectively.

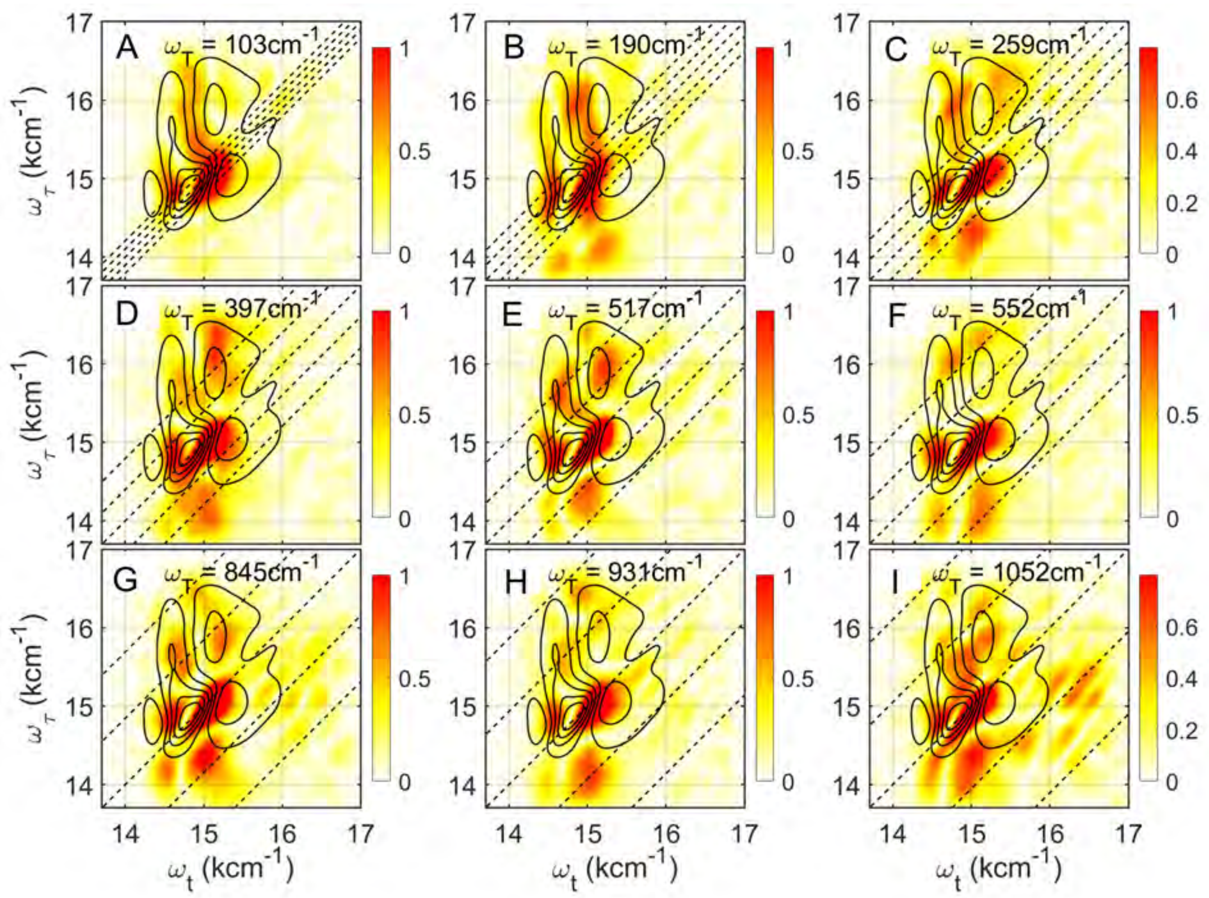

FIG. S9: The 2D vibrational maps with selected frequencies are shown form A to I. The black dashed lines indicate the vibrational progression of the modes.

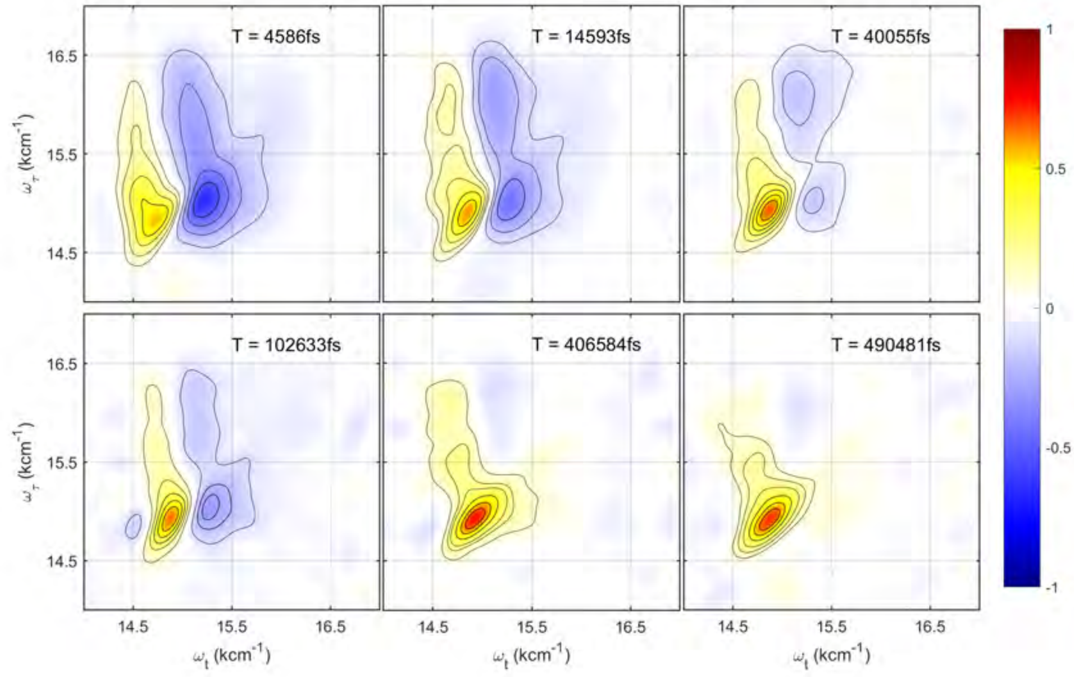

FIG. S10: The measured 2DES with selected waiting time up to 500 ps. The measuring temperature is 80 K.

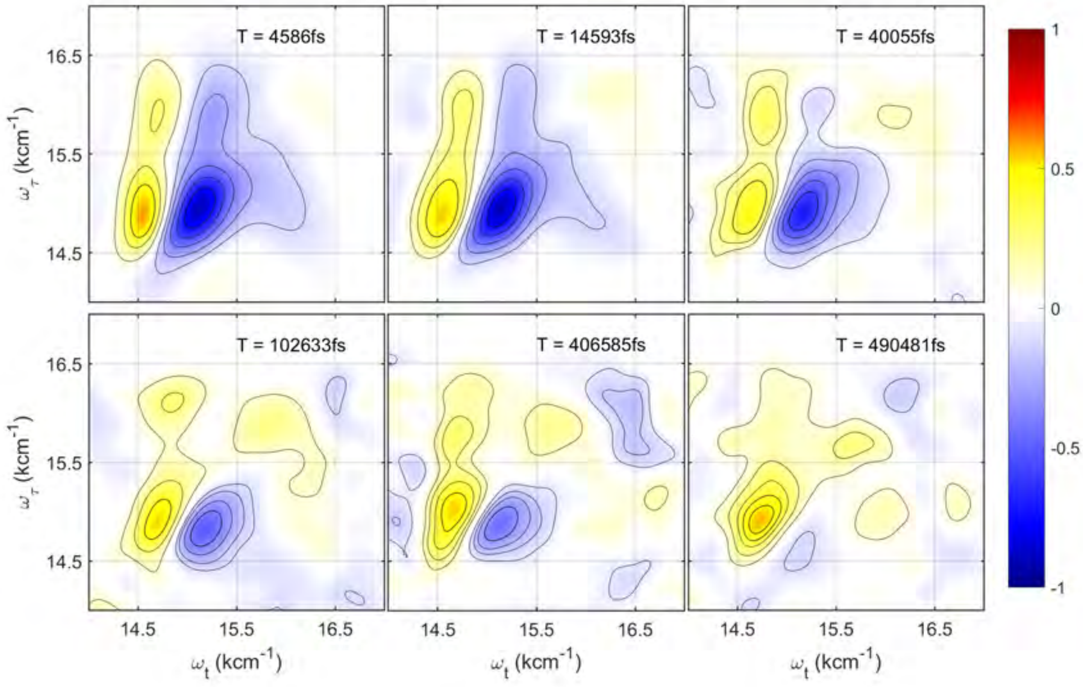

FIG. S11: Measured 2DES with selected waiting time up to 500 ps. The measuring temperature is 8 K.

TABLE I: **Matrix elements of the CT Hamiltonian in basis states. All entries are given in units of cm^{-1}.**

	$ec_2A^+ec_3A^-$	$ec_2B^+ec_3B^-$	$P_{700}^+ec_3A^-$	$P_{700}^+ec_3B^-$
$ec_2A^+ec_3A^-$	14000	0	50	0
$ec_2B^+ec_3B^-$	0	14030	0	40
$P_{700}^+ec_3A^-$	50	0	13730	0
$P_{700}^+ec_3B^-$	0	40	0	13750

TABLE II: **Fitting parameters of calculated population dynamics at different temperatures. The dynamics were fit to provide the effective 1/e decay times for each component. All entries are given in units of fs.**

	**Lhcr1**	**Lhcr2**	**Lhcr3**	**PsaA**	**PsaB**
300 K	b1=228<br>b2=10102<br>b3=70349	b1=2809<br>b2=10867	b1=627<br>b2=4811	b1=2882<br>b2=9040	b1=2873<br>b2=11707<br>b3=39712
77 K	b1=136<br>b2=16947<br>b3=17187	b1=1811<br>b2=24494	b1=732<br>b2=4186<br>b3=24967	b1=1079<br>b2=8450<br>b3=36201	b1=9473<br>b2=18503
40 K	b1=95<br>b2=20997	b1=997<br>b2=1065<br>b3=71488	b1=349<br>b2=4340<br>b3=105730	b1=956<br>b2=7559<br>b3=50641	b1=326<br>b2=6961<br>b3=35691
20 K	b1=50<br>b2=20432	b1=572<br>b2=407<br>b3=615<br>b4=472308	b1=1149<br>b2=1206<br>b3=1687<br>b4=1.24213e8	b1=599<br>b2=4254<br>b3=35263	b1=2795<br>b2=3608<br>b3=1.49362e8

TABLE III: **Fitting Parameters of population dynamics at different temperatures obtained from experimental 2DES data. The dynamics were fit to provide the effective 1/e decay times for each component. All entries are given in units of fs.**

	**1**	**2**	**3**	**4**	**5**
80 K	885	4257	18459	537984	Inf
8 K	383	2650	53871	265385	Inf
